# Temperature–Pressure Swing Process for Reactive Carbon Capture and Conversion to Methanol: Techno-Economic Analysis and Life Cycle Assessment

**DOI:** 10.1021/acs.est.4c02589

**Published:** 2024-07-24

**Authors:** Jonathan A. Martin, Eric C. D. Tan, Daniel A. Ruddy, Jennifer King, Anh T. To

**Affiliations:** National Renewable Energy Laboratory (NREL), 15013 Denver West Parkway, Golden, Colorado 80401, United States

**Keywords:** methanol, hydrogen, carbon capture, reactive carbon capture, flue gas, techno-economic analysis, life cycle assessment, decarbonization

## Abstract

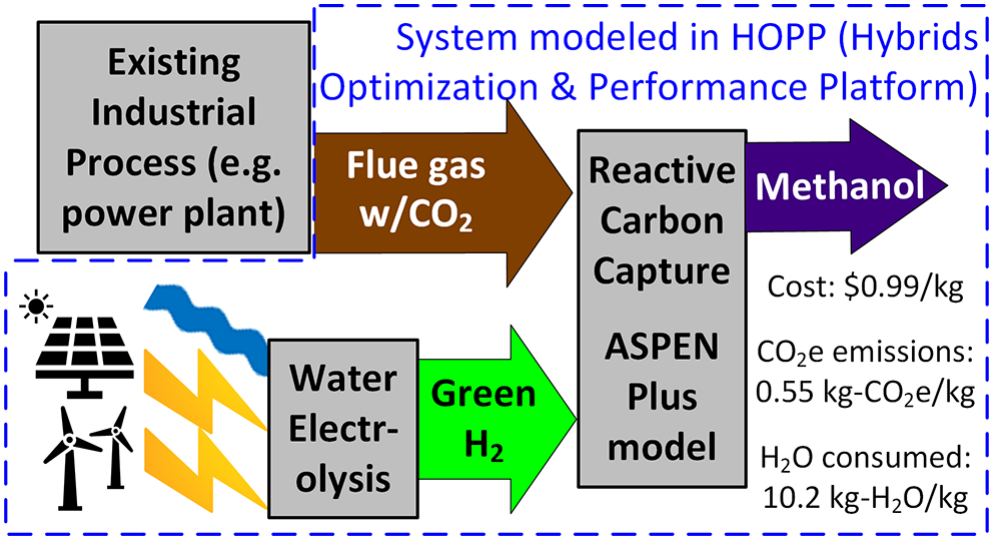

A model was developed to conduct techno-economic analysis (TEA) and life cycle assessment (LCA) for reactive carbon capture (RCC) and conversion of carbon dioxide (CO_2_) to methanol. This RCC process is compared to a baseline commercialized flue gas CO_2_ hydrogenation process. An ASPEN model was combined with existing TEA and LCA models into a larger TEA/LCA framework in Python. From preliminary experimental data, the model found a levelized cost of $0.79/kg methanol for the baseline process and $0.99/kg for the RCC process. The cradle-to-gate carbon intensity of the baseline process was 0.50 kg-CO_2_e/kg-methanol, compared to 0.55 kg-CO_2_e/kg-methanol for the RCC process. However, water consumption for RCC (10.21 kg-H_2_O/kg-methanol) is greatly reduced compared to the baseline (12.89 kg-H_2_O/kg-methanol). Future improvements in hydrogen electrolysis costs will benefit the RCC. A target H_2_/methanol mass ratio of 0.26 was developed for RCC laboratory experiments to reduce methanol cost below the baseline. If a ratio of 0.24 can be achieved, a levelized cost of $0.76/kg methanol is projected, with a carbon intensity of 0.42 kg-CO_2_e/kg-methanol.

## Abstract 2

Carbon emitted to the atmosphere by industry can be reduced by developing chemical pathways from raw industrial flue gas to valuable products like methanol, without the need to interrupt the industrial process to install carbon capture and purification equipment.

## Introduction

Although many countries worldwide share the goal of eventually shifting to fully decarbonized energy systems, these systems are not developing at the rates needed to fully replace fossil fuel combustion systems before climate change reaches critical levels. To achieve CO_2_ reduction goals, combustion systems must be equipped with carbon capture, utilization, and storage (CCUS) systems.^1^ Carbon capture and storage (CCS) is already commercially available to capture CO_2_ emissions, but it is not widely implemented due to the high capture cost and lack of economic benefits of the captured CO_2_.^2^ A more feasible alternative to CCS is carbon capture and utilization (CCU), which not only captures the CO_2_ emissions but also converts them into valuable chemicals and fuels, offsetting the cost of capture and adding economic value to the decarbonization process.^2,3^ A reactive carbon capture (RCC) approach, where CO_2_ capture and conversion processes are integrated to avoid the costly CO_2_ desorption and capture media regeneration steps, can help minimize the costs, energy usage, and water consumption (WC) requirements of CCU.^4,5^

Our research group recently reported the development of dual function materials (DFMs) and the associated temperature and pressure swing RCC process to produce methanol. Conceptual operation of an RCC methanol production system involves a two-step process using multiple temperature and pressure swing reactors (TPSRs) in pairs. In the first step, combustion flue gas fills the first TPSR (R1) at low pressure where CO_2_ is adsorbed onto a DFM bed. In the second step, the flue gas is redirected to the second TPSR (R2) while the first TPSR (R1) is pressurized with hydrogen, and the temperature is ramped up to 250 °C, simultaneously regenerating the catalyst and reacting with the adsorbed CO_2_. The detailed chemistry of this process is discussed by Jeong-Potter et al. in a recent experimental study.^6^

To assess the economic and environmental viability of the RCC approach for methanol production, it is important to develop a process model that can extrapolate from laboratory-scale results to commercial-scale production for the RCC approach and compare with methanol production from the conventional CO_2_ hydrogenation approach. The goal of this study is thus to establish a framework for both techno-economic analysis (TEA) and life cycle assessment (LCA) that will compare the two routes of CO_2_ hydrogenation (the baseline method) and RCC for producing methanol from CO_2_ and renewable hydrogen. This will help establish whether the RCC method is worth further development toward a commercial CCU process that can effectively contribute to CO_2_ reduction targets.

The production of low-carbon methanol fills several gaps in the low-carbon economy that have not been filled. Methanol can be used as a fuel, especially in the shipping industry, which is particularly difficult to decarbonize through electrification.^7^ Shipping giant Maersk has recently made this a reality by signing a deal with green methanol producer OCI Global to prepare for the maiden voyage of a methanol-enabled container ship, while committing to transport a minimum of 25% of its ocean cargo using green fuels by 2030,^8^ signaling a growing need for low-carbon marine fuels that methanol is ideally suited to fill. Besides its fuel applications, methanol is used to produce other chemicals such as formaldehyde, acetic acid, and plastics, and nearly all of its current 98 million tonnes per annum production currently comes from fossil fuels.^9,10^ Currently, most green methanol is derived from renewable feedstocks via biomass-to-methanol routes. However, the production of “biomethanol” is subject to biomass feedstock availability constraints, whereas CO_2_-derived methanol production pathways do not face this feedstock limitation. Therefore, a low-carbon CO_2_-to-methanol process that is economically competitive with fossil fuel methanol production routes is urgently needed if methanol is to play a significant role in decarbonization.

There are commercialized processes to convert CO_2_ and renewable H_2_ to methanol, namely, carbon capture and purification followed by CO_2_ hydrogenation.^11,12^ Hydrogen cost (via renewable electricity cost and capacity factor) has been identified as the most critical factor toward the economic viability of this pathway.^13^ The sourcing of H_2_ (via the source of electricity that powers electrolysis) is also the most critical factor in the overall environmental impact of the production of this type of methanol, giving it a wider range of estimates for its CO_2_ emissions than those of other renewable methanol routes. The first successful commercial plant was established in Iceland in 2011, where geothermal plants can supply ample CO_2_ and electricity for H_2_ electrolysis, and the largest operating plant at 100 000 t/yr methanol was commissioned by the same company in October 2022 at a coke production facility in Anyang, China.^14^ However, most, if not all, of the publicly available economic and environmental data on this and similar processes is currently in the form of modeling studies.^9^

## Materials and Methods

**Figure 1:**
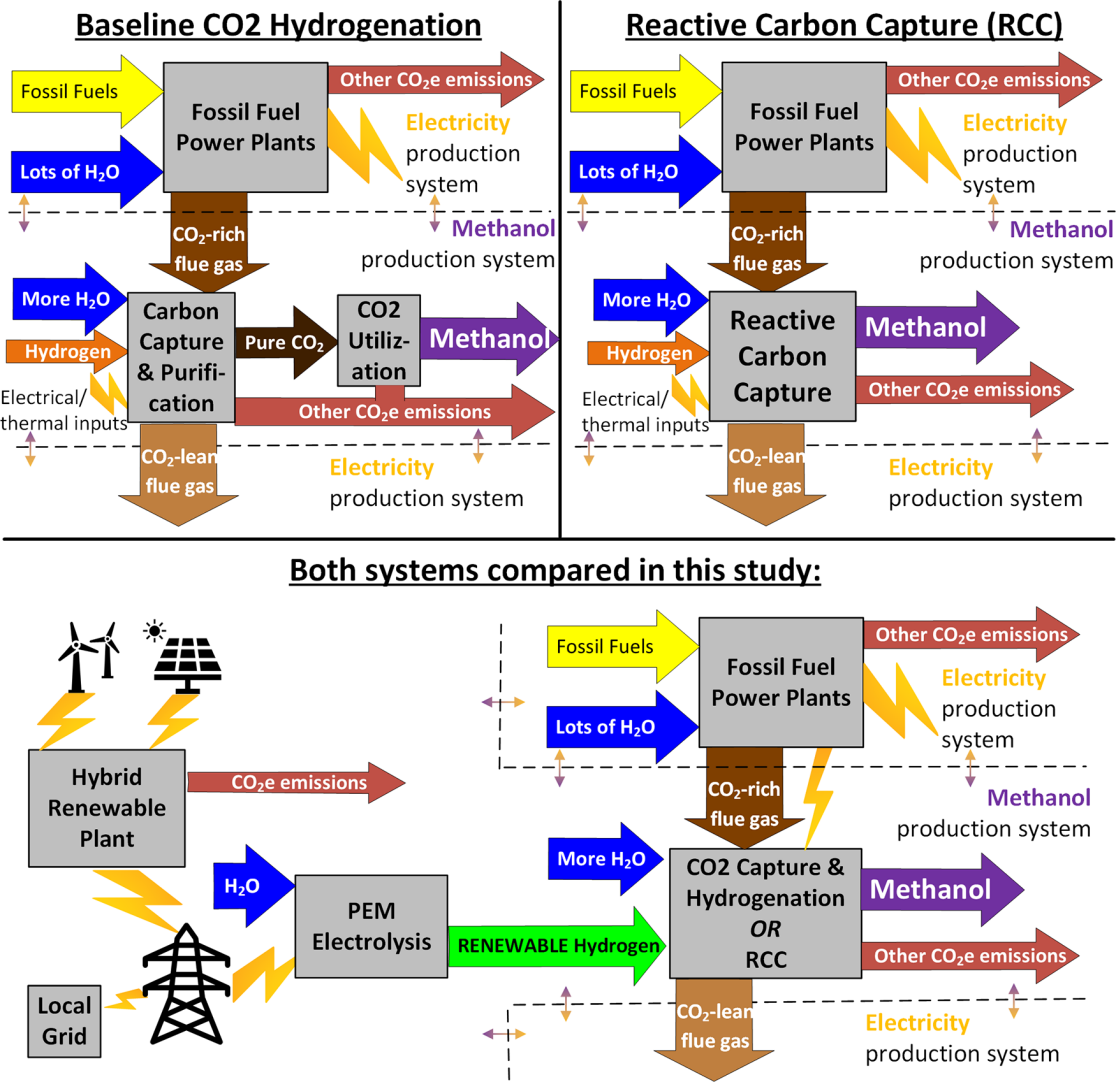
Comparison of baseline CO_2_ hydrogenation and RCC approaches with system boundaries for TEA/LCA of electricity and methanol production.

A schematic comparison of the baseline CO_2_ hydrogenation and RCC approaches is shown in Figure 1. This figure shows how both approaches create two product streams, one each with its own system boundary that can be used for both a TEA and an LCA. In both cases, the system boundary for the production of methanol is drawn within the flow of flue gas out of the fossil fuel power plant. This breaks the TEA and LCA into two parts, one for the production of electricity and the other for the production of methanol.

The TEA/LCA process model developed in this study will compare two CO_2_/H_2_-to-methanol processes:

1. The baseline CO_2_ hydrogenation process, representing the state of the art for methanol CCU.
2. The RCC process using reference dual function material (DFM), i.e., CZA, performance developed by NREL.^6^

Both of these processes will consist of three major steps:

1. Production of CO_2_, which will be from the same source for both processes but have different purity requirements for each.
2. Production of H_2_, which will be identical in source and purity for both processes but will be required in different amounts per unit methanol for each.
3. Production of methanol from CO_2_ and H_2_, which will be analyzed using literature models for the baseline process, and the NREL-developed ASPEN plus model for the RCC process.

For the baseline process, CO_2_ must be captured, separated, purified, and compressed through a relatively expensive purification-desorption step prior to feeding to hydrogenation reactor. In contrast, for the RCC process, dilute CO_2_ that has not been purified can be used directly. To keep the comparison as “apples-to-apples” as possible, both processes were modeled using the same CO_2_ source: a natural gas combined cycle (NGCC) power plant. Natural gas has continually grown its share of the world electricity market in recent decades even as renewables have risen and other fossil fuels have declined,^15^ making it one of the most important targets for CCU application development.

Given that both processes aim to produce a product with low net carbon intensity (CI), the hydrogen used as a feedstock must be produced with as low a carbon intensity as possible. However, producing renewable hydrogen while maintaining low production costs has proven to be a significant technological challenge. A very promising route, with many pathways to future cost reductions through research and development, is proton exchange membrane (PEM) water electrolysis.^16^ While the cost of PEM-based hydrogen can be driven down by cheaper electricity, and renewable electricity costs per kWh have begun to eclipse fossil fuel electricity costs, the low capacity factors of renewable plants make it challenging for these plants to power hydrogen electrolysis for a low resultant price of hydrogen.^17^ One potential solution to this problem is to power electrolysis with a hybrid renewable plant, specifically a hybrid wind/solar plant that is able to take advantage of wind/solar resource complementarity to provide a more reliably constant power supply.^18^ While the specific site for such a hybrid will depend on many things, such as land availability, this study specifically focuses on building methanol RCC capability onto existing NGCC plants. As such, we focused on sites within 100 km of existing large (>200 MW) NGCC plants, so that costs of electricity transmission and/or hydrogen transport from the generation site to the methanol production site (on-site at the NGCC plant) could be considered negligible.

In converting CO_2_ and H_2_ to methanol, three literature TEA studies were available for the baseline process.^19−21^ Average values from these studies were used as inputs to the process model. For the baseline process LCA, inputs for CI were taken from Adnan and Kibria,^22^ while water consumption (WC) inputs were taken from Nyari et al.^21^ For the RCC process, an ASPEN Plus reactor model was developed by using NREL experimental data as inputs. This ASPEN model was detailed in the Materials and Methods, and provides production cost, CI, and WC inputs to the overall process model. The main metric of the LCA is the CI of the overall process, calculated by summing the CI of the subprocesses, while the main metric of the TEA is the Levelized Cost of Methanol (LCOM), expressed in 2020 dollars per kilogram of methanol produced.

To determine the sensitivity of the RCC technology’s LCOM and CI to changes in the modeled scenario, three types of parameter sweeps were conducted:

1. Reactor performance, i.e., the ability of the RCC process to efficiently convert CO_2_ and H_2_ to methanol without leftover reactants and secondary products requiring separation.
2. Start-up year, i.e., the year from which pricing assumptions are taken for model inputs in which prices vary with time. This allows for projections of reduced costs of renewable hydrogen in future years.
3. Location, i.e., the specific NGCC plant at which the methanol reactor will be built and near which the hybrid wind/solar/H_2_ plant will be constructed.

All of these parameter sweeps begin from a pair of starting test cases, which are based on preliminary RCC performance of a DFM reported elsewhere^6^ and real-world results in the literature, not a projected or modeled scenario. The parameters and sources for these starting test cases are given in the following section and Supporting Information.

### Python TEA/LCA Model

The outfitting of NGCC plants with carbon capture systems has been extensively studied by the National Energy Technology Laboratory (NETL), including calculations of the breakeven sales price of CO_2_ (or tax on CO_2_ emissions) necessary to support carbon capture installations,^23^ which will be considered as the CO_2_ production cost for the baseline process. NETL also carried out a full LCA of a baseline plant with and without carbon capture that was used as input data to the process model in this study.^24^ In our process model, the equivalent CO_2_ emissions (CO_2_e) of the NETL plant without carbon capture are attributed to electricity, and the additional CO_2_e emissions of the plant with carbon capture (the difference between the two plants) are attributed to the purified CO_2_ product. It should be noted that the CO_2_e emissions calculated by the LCA (i.e., the carbon intensity of CO_2_ capture) and the purified CO_2_ sent to methanol production are two separate, nonoverlapping entities since this purified CO_2_ will be consumed later in the process and not emitted to the atmosphere.

For the baseline process, CO_2_ is captured and purified from the NGCC flue gas. For the RCC process, CO_2_ does not need to be purified and can be taken directly from the NGCC flue gas. Thus, in the process model, the production cost, carbon intensity (CI), and WC of the CO_2_ going into the methanol process were set to zero since these costs could be directly attributed to electricity generation instead (see Figure 1).

NREL has developed a tool to design an integrated, grid-connected hybrid wind/solar/PEM plant that is optimized to reduce hydrogen production costs at a given site, called the Hybrids Optimization and Performance Platform (HOPP).^25^ HOPP-modeled hybrids are used to determine the production cost and carbon intensity of H_2_ needed for both methanol processes. As inputs to HOPP, the cost of the hybrid plant was determined from NREL’s Annual Technology Baseline (ATB),^26^ while the cost of buying and selling grid electricity was modeled from NREL’s Cambium model,^27^ the US Energy Information,^28^ and LBNL power purchase agreement (PPA) data.^29,30^ CI and WC of the overall electricity mix were determined from the NETL Grid Mix Explorer^31^ for the wind and solar production and from NREL’s Cambium model for electricity purchased from/sold to the grid.^27^ A scalable CI of the PEM electrolyzer stack was taken from a study by Zhao et al.,^32^ while the WC required by the electrolyzer, as well as its production cost, was determined from NREL’s own Hydrogen Analysis (H2A) case studies.^33^ These inputs are detailed further in the Supporting Information.

The levelized cost of methanol (LCOM) represents the average amount of revenue required over the plant’s operational life to meet all capital and operations costs and does not match the final selling price of methanol required to generate a profit margin. LCOM, along with other levelized costs calculated in parts of the model (such as levelized cost of electricity or LCOE), use the simple fixed-charge rate (FCR) method used by NREL’s System Advisor Model (SAM),^34^ the financial model used in HOPP.
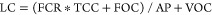
1

The terms in eq 1 are

- LC: Levelized cost [$/MWh, for energy OR $/kg, for materials]
- TCC: Total capital cost [$]
- FOC: Fixed operating cost [$/yr]
- AP: Annual production [MWh/year, for energy OR mt/year, for materials]
- VOC: Variable operating cost [$/MWh, for energy OR $/mt, for materials]

Further details on the financial calculations can be found in the Supporting Information.

### Reactive Carbon Capture Conceptual Process Design

The conceptual process models for the RCC process were developed in Aspen Plus. The model areas include CO_2_ adsorption, hydrogen compression, and hydrogenation reaction as well as methanol separation, steam generation, and utilities. Additionally, the process incorporates renewable H_2_ and utilizes RCC experimental performance data over a Cu–Zn–Al mixed oxide (CZA) catalytic material. The RCC annual plant capacity was set at 115 104 tonnes. The flue gas flow from a natural gas power plant was assumed to contain 10 wt % CO_2_ and was adjusted and fed to the process. The flue gas CO_2_ feed to the RCC reactor was at 4110 million metric tonnes per year (MMt/year) for the case without partial recycling of product stream. For the case with the reactor effluent recycling, the flue gas could be reduced to 1050 MMt/y to produce the same amount of methanol product (due to high overall CO_2_-to-methanol conversion efficiency). The CO_2_ adsorption efficiency was assumed to be 20%; the CO_2_ adsorption capacity (66 μmol/g-catalyst) and single-pass methanol productivity (15.77 μmol/g-catalyst) used in the model were obtained from experimental RCC performance of CZA material.^6^ For the case with recycle, the resulting overall methanol productivity was calculated to be 68.13 μmol/g-cat. The net hydrogen consumption was determined to be 1.00 kg H_2_/kg methanol without recycle and 0.33 kg H_2_/kg methanol with recycle.

### Parameter Sweeps

In each of the parameter sweeps given below, only one parameter was varied. All other parameters were held constant to the “starting test case” with recycling of end gas, with parameters detailed in Table S1 of the Supporting Information.

To determine the sensitivity of LCOM and CI to reactor performance, three sweeps were performed. These sweeps model the effects of improved reactor performance on the LCOE and CI, to determine the improved performance achievable if improved experimental results are obtained. The sweeps are the following:

1. Catalyst CO_2_ adsorption capacity. This parameter reflects the efficiency with which the flue gas CO_2_ is captured from the flue gas in the first step of the RCC process described in the Introduction section. It was swept from 66 μmol/g-catalyst (the result from preliminary experiments) to 150 μmol/g-catalyst.
2. MeOH selectivity during reactive desorption. This parameter is the efficiency with which the captured CO_2_ reacts to form methanol as opposed to other products (e.g., methane and CO) when the reactor is pressurized with H_2_ in the second step of the RCC process described in the Introduction section. It was swept from 23% (the result from preliminary experiments) to 50%.
3. Hydrogen consumption per unit methanol. This is the overall ratio of hydrogen consumed to methanol produced, which is critical to make as low as possible for an economically competitive process, given the expense of renewable hydrogen. This parameter is dependent on the methanol selectivity and will also vary during sweep #2. However, during sweep #3, methanol selectivity was held constant while H_2_ consumption was swept, to model more efficient hydrogen utilization. The H_2_ ratio was swept from 0.33 down to 0.2, as seen in Figure 3.

Only the H_2/_methanol ratio sweep is shown in the main body of the paper, with the rest of the results given in the Supporting Information due to space constraints. These reactor performance sweeps do not necessarily reflect the range of results that are expected to be achievable with this technology, rather they are being used to develop targets; i.e., they will determine what values of each parameter will need to be achieved by RCC for it to be competitive with CO_2_ hydrogenation as a methanol production process.

In addition to the reactor performance sweeps, sweeps of the start-up year and location were performed. Start-up year of the modeled methanol plant was swept from 2020, in which measured data on electricity pricing was available for the HOPP input models, in 5 year increments out to 2050. This allowed for modeling of improvements in renewable energy technologies that reduced not only the HOPP-modeled price of renewable H_2_ but also the CI of the electricity that produced it.

## Results and Discussion

The results of the TEA and LCA for the RCC process will be broken down into four sections, starting with a first section “Starting Test Cases” which is based purely on the experimental results and followed by one section each on the three-parameter sweeps, namely, reactor performance, start-up year, and location.

### Starting Test Cases

**Figure 2:**
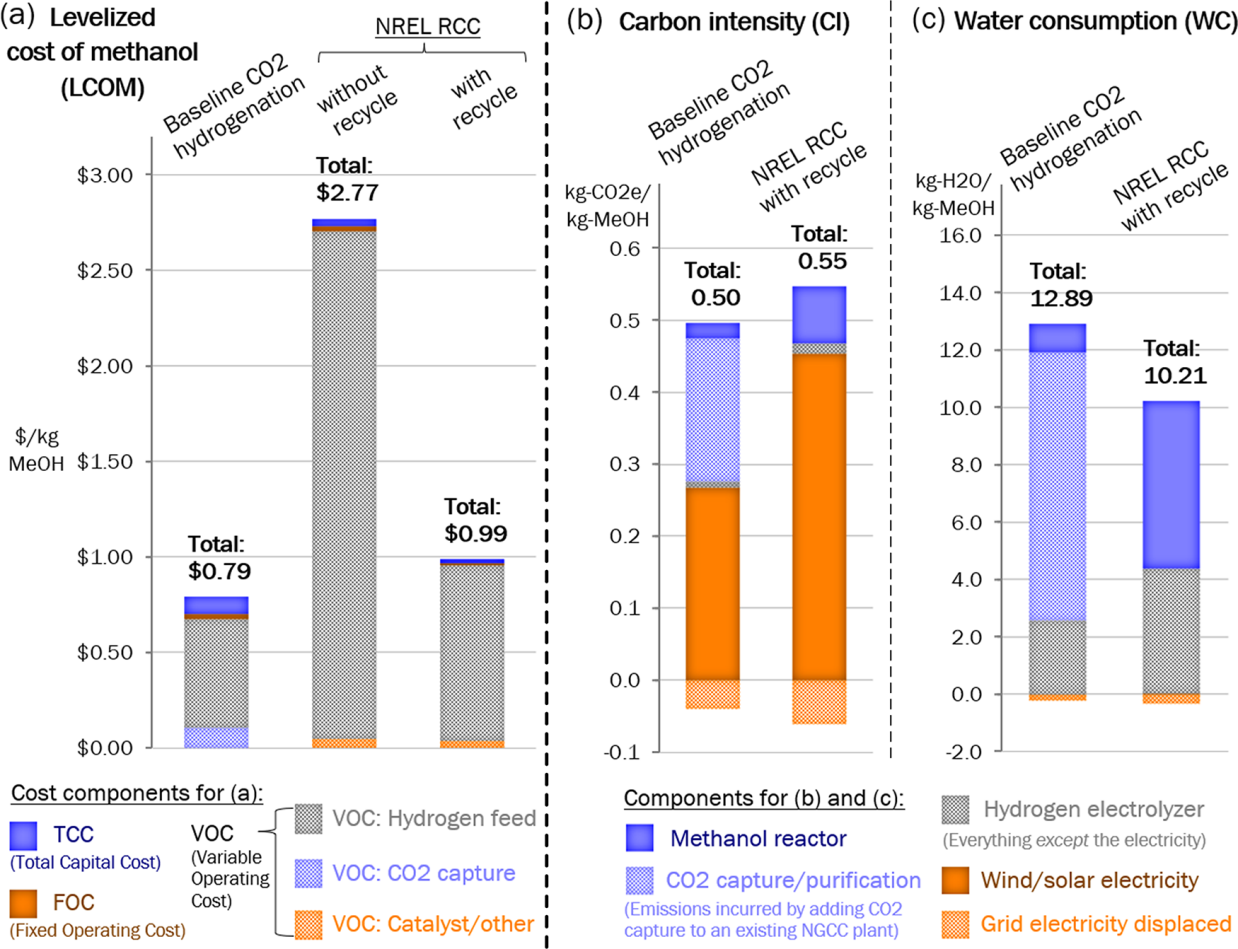
TEA/LCA results are given with breakdowns for starting test cases.

The starting test cases were fed into the process model and compared to the baseline CO_2_ hydrogenation process, with TEA results given in Figure 2(a) and the LCA results given in Figure 2(b,c). Since the RCC process without recycle (e.g., unconverted H_2_, intermediate product CO) produced an LCOM more than three times the baseline, the nonrecycle case is excluded from the following results, and all results from here out are for the case with end gas recycling.

With end gas recycling, the initial experimental TEA results are much more promising, with the LCOM of the RCC process (0.99 $/kg of MeOH) starting out only 25% more expensive than the baseline process (0.79 $/kg-MeOH). As seen in the breakdowns of this cost, most of the production cost in both cases comes from producing hydrogen, but even more so with RCC. With RCC, the variable operating cost of H_2_ production is 96% of the total LCOM, whereas in the baseline technology, it is only 82%. The baseline technology has higher expenses for total capital cost (i.e., building the methanol synthesis reactor) and the capture and purifying CO_2_. Similarly, most of the carbon emissions from both processes are coming from the electricity used to generate the hydrogen, and more so for the RCC than the baseline, leading the RCC to have a slightly higher overall CI by 10%.

However, a major benefit of the RCC process is that it consumes 21% less water than the baseline. Unlike the other two breakdowns, the WC breakdown is dominated by a different subprocess for each of the two methanol production routes. In the baseline process, CO_2_ capture and purification consumes the most water, almost as much as the entire RCC process. In the RCC process, the methanol synthesis reactor itself consumes the most water. The RCC process direct water consumption (1.51 gal/kg MeOH) is attributed to cooling tower makeup and boiler feedwater (BFW) makeup, 23 and 77%, respectively.

### Reactor Performance Sweep

**Figure 3:**
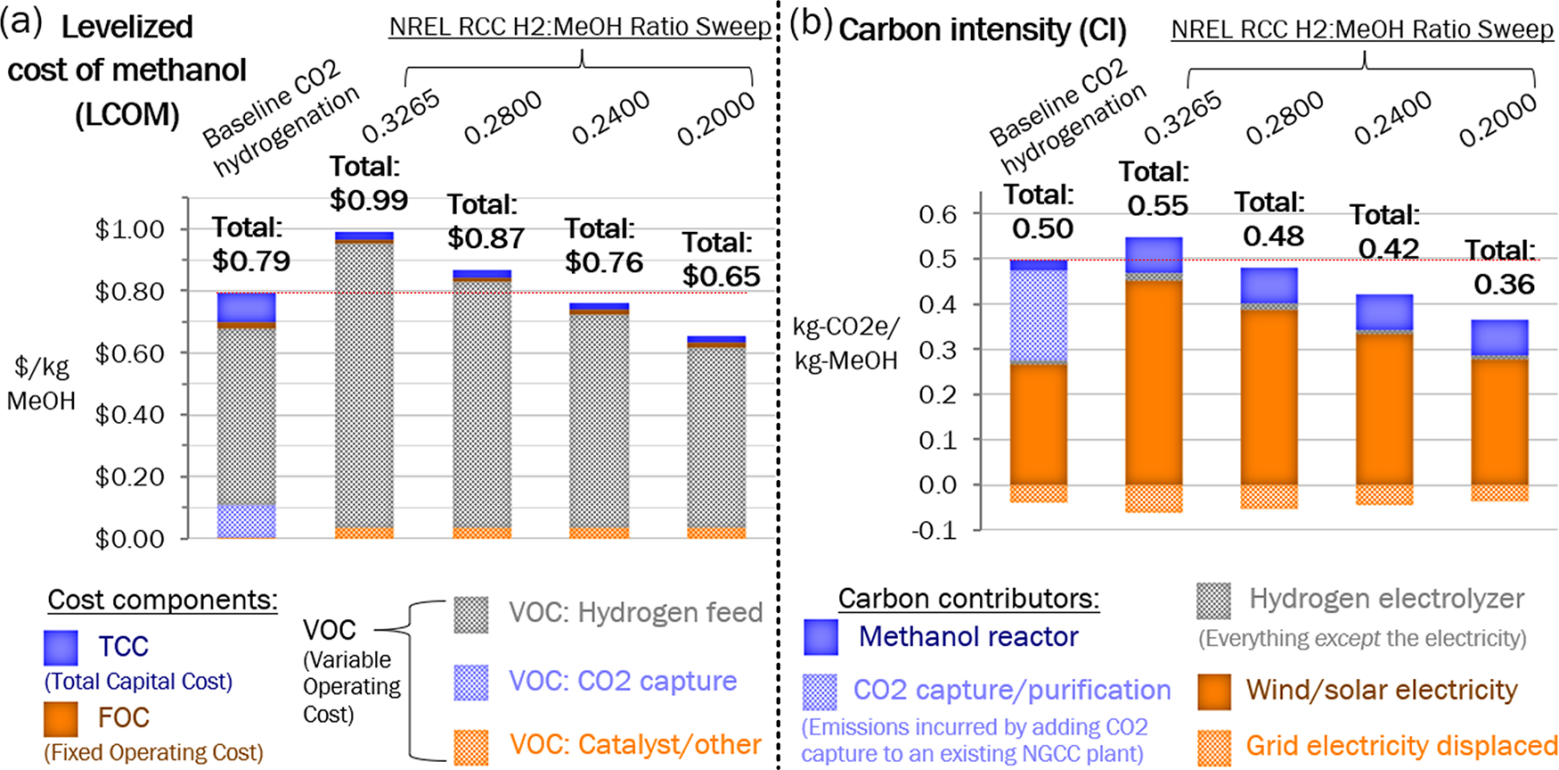
TEA and LCA results with breakdowns for H_2_/Methanol ratio sweep.

To investigate potential cost reductions due to the high cost of renewable H_2_ in the RCC process, a sweep of methanol reactor performance in terms of the mass ratio of hydrogen used to methanol produced is shown in Figure 3. The H_2_:methanol ratio was not directly measured from the RCC experiments, but rather, the ratio was modeled in ASPEN as a result of these experiments, which are detailed in our recent publication.^6^ To illustrate why the H_2_:methanol ratio is so critical to reducing LCOM and CI, breakdowns of these metrics are shown in Figure 3. When the H_2_/methanol ratio reaches 0.24, the H_2_ production component of both LCOM and CI is still greater than the baseline, but because the other components of RCC are so much smaller, the overall LCOM and CI are both lower than the baseline by this point. If an H_2_/methanol ratio equivalent to the baseline ratio of 0.20 could be achieved, these benefits would be even more substantial.

### Start-Up Year Sweep

**Figure 4:**
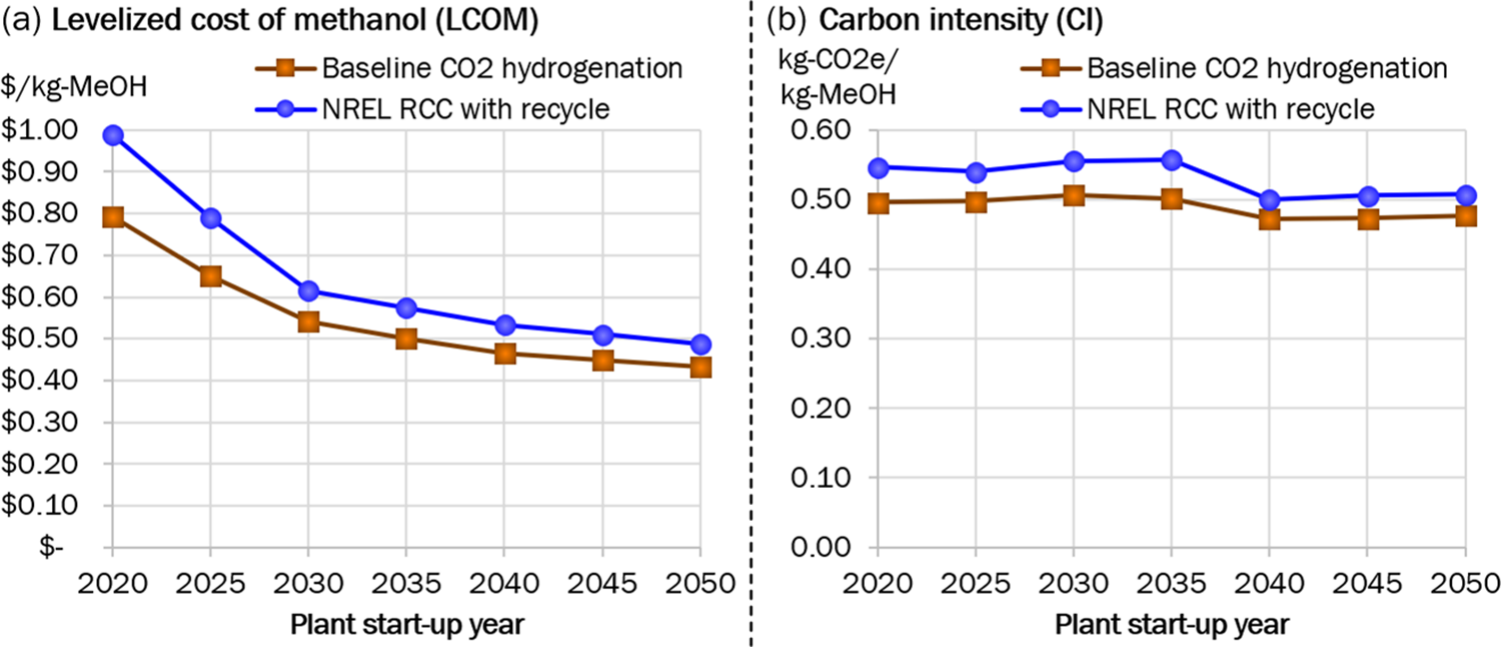
TEA and LCA results for start-up year sweep.

Projected changes in LCOM and CI with time, as nascent technologies are projected to mature, are shown in Figure 4. The primary driver of the LCOM reductions is the projected reduced costs of renewable electricity used to power electrolysis, which, in turn, is projected to have lower capital costs as PEM technology matures in the coming decades. Since H_2_ electrolysis costs via renewable electricity are a larger portion of the RCC cost than the baseline cost, these cost reductions narrow the gap between the baseline technology and RCC.

**Figure 5:**
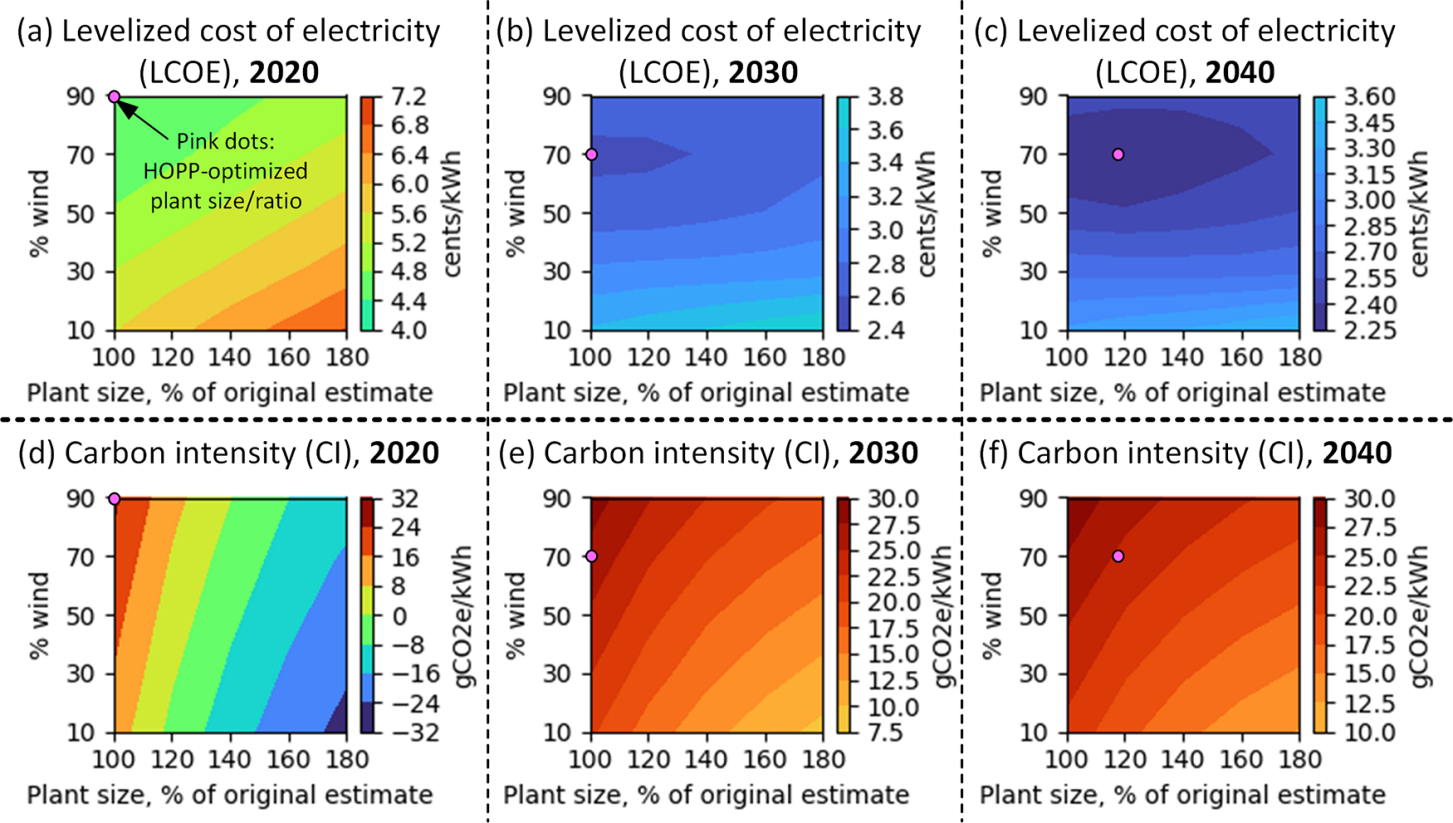
Optimization of wind/solar hybrid plant design using HOPP over different plant start-up years. The top row (panels a–c) shows LCOE while the bottom row (panels d–f) shows CI. Each panel has its own color bar. Both LCOE and CI are the combination of wind/solar electricity going to hydrogen electrolysis plus grid electricity displaced–the total contribution of the plant to the overall TEA/LCA (see Figures 1, S6, and S7).

To understand the shape of the trend in CI, it is useful to understand the process by which HOPP optimizes the design of the wind/solar hybrid plant, a process that is shown in Figure 5. HOPP minimizes the LCOE for the wind/solar hybrid plant for the given start-up year, iterating through different ratios of wind vs solar and overall output capacities. HOPP models the plant buying and selling electricity from the grid to keep the electrolyzer near full capacity and the losses associated with the gaps between the costs of renewable electricity and the power purchase agreements negotiated for these plants, as described in the Methods and Materials section and further in Supporting Information. In 2020, the optimal plant was found at the edges of bounds that HOPP was optimizing, with a maximum of 90% wind and a minimum of 100% of the originally estimated plant size, which meant that the hybrid was just large enough to supply electricity to H_2_ electrolysis on average. The CI of the overall electricity mix in 2020 was thus very similar to the CI of wind energy alone. A larger wind/solar hybrid would have displaced much more grid electricity (potentially even making the entire plant carbon-negative on the whole), but this would be too expensive to consider.

Moving out to the year 2030, solar costs are projected to become closer to wind. This moves the optimal wind/solar ratio away from the optimization bounds. Moving further out to the year 2040, it begins to become economical to “overbuild” the hybrid plant as wind/solar prices become competitive with grid electricity. When this line is crossed, the wind/solar hybrid starts becoming a substantial net exporter of electricity and a substantial net displacer of grid CO_2_ emissions. This explains the “kink” in the CI graph seen in 2040 in Figure 4. Since electricity forms a larger share of the overall CI breakdown for the NREL RCC than for the baseline CO_2_ hydrogenation (see Figure 2b), the shift to overbuilding and the associated reduction in CI is greater for RCC. This causes the CI of the two to become closer together starting in 2040 in Figure 4.

There are three caveats to this analysis:

1. This is based on current projections of grid carbon emissions, and if efforts to reduce the carbon intensity of the grid are successful, then a net exporter of electricity would not be a net displacer of CO_2_ emissions.
2. This analysis assumes that grid prices are fully elastic, and there is no internalized cost from the stress put on the grid by the hybrid plant buying and selling electricity as needed to maintain full capacity at the electrolyzer.

However, these are issues that apply to broader decarbonization efforts, in general, and are outside the scope of this study.

### Location Sweep

**Figure 6:**
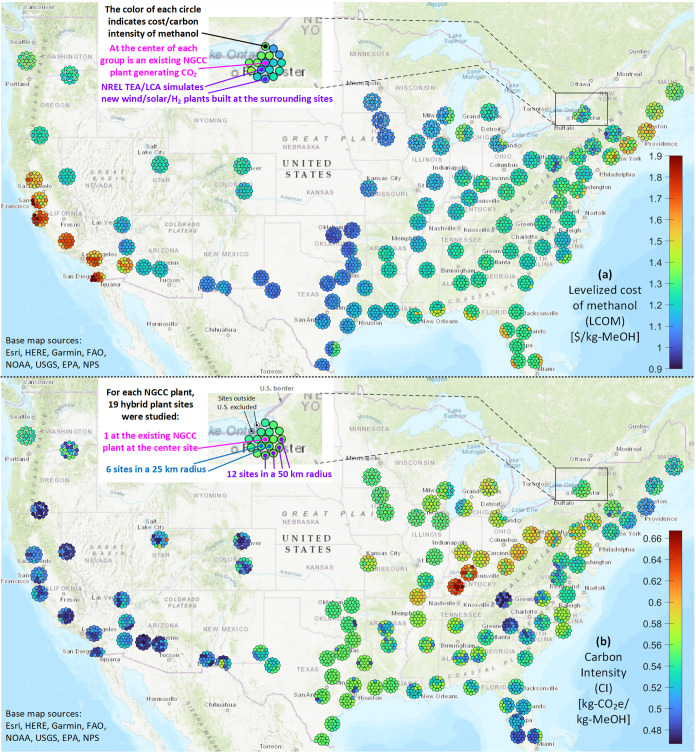
TEA/LCA results from the location sweep across the USA. (a) Variations in the LCOM. (b) Variations in CI. The base map was created using ArcGIS software by Esri. Copyright Esri.

The same optimization of wind/solar plants was performed at many locations near existing NGCC plants (described further in the Materials and Methods section) to produce a minimized LCOM for each location, as shown in Figure 6(a). This illustrates how the “wind corridor” locations from Texas up to Iowa are currently where this methanol production technique is most economical given the best natural wind resources for electricity-based H_2_ production. However, prices are not prohibitively more expensive in other locations such as the southeast and mountain west. The main reason for the price gap in these locations is that the ideal hybrid plant design features more solar than wind, and current HOPP modeling with NREL Annual Technology Baseline (ATB) prices^26^ put a slightly higher price on utility-scale solar PV electricity than land-based wind electricity. Meanwhile, offshore wind is still a developing technology and shows an approximately $0.30 price gap with nearby onshore methanol production sites but has fewer space constraints than land-based wind.

The areas where RCC methanol production is most limited are where grid electricity prices are the highest, particularly in Massachusetts and California. Here, a pure wind/solar hybrid may not be the best solution, and methanol production costs could be reduced by employing energy storage to reduce the dependence on the grid. This could take the form of a battery energy storage system to store excess wind and solar generation and discharge it to the electrolyzer when needed. Alternatively, a hydrogen storage system could store excess generation in the form of hydrogen and be combined with a fuel cell to send electricity back to the electrolyzer when it is needed. But again, this issue of grid energy storage is a broad decarbonization problem that is outside the scope of this study.

When hybrid plant design is optimized for minimal LCOM, it does not minimize CI, as Figure 6(b) shows. The lowest-LCOM plants in the “wind corridor” tend to have CI in the middle of the range of all of the plants studied in the location sweep. This is due to the higher CO_2_e emissions of wind electricity (29.5 kg-CO_2_e/MWh) relative to solar PV electricity (22.4 kg-CO_2_e/MWh) in this model, taken from the NETL Grid Mix Explorer.^31^ For this reason, lower CIs are found in sunnier areas, such as Florida and the Southwest where hybrid plant output is optimized by a higher proportion of solar PV to wind. The CO_2_e emissions of these renewable generators pale in comparison to the grid mix of coal-heavy areas such as Kentucky, where the average CI of grid electricity is currently 944.6 kg-CO_2_e/MWh according to NREL’s Cambium model.^27^ In these areas, the CI of methanol is highly dependent on whether the optimal hybrid plant wind/solar plant design is a net buyer or seller of grid electricity. If there are low wind/solar resources and the wind/solar plant must buy more grid electricity than it sells to keep the H_2_ electrolyzer running at a high capacity, this will have a detrimental impact on the overall CI of the methanol.

## Discussion

To make methanol produced by RCC cheaper and lower carbon intensity than the baseline, performance objectives for catalyst development must be developed. The reactor performance sweep in Figure 3 identified the ratio of hydrogen consumption to methanol production as a key performance objective. This ratio is only 0.20 in the baseline process, whereas it is 0.33 in the initial process modeling results. A ratio of 0.26 would bring the LCOM of the RCC process equal to the baseline while bringing the CI lower than the baseline; therefore, this is the target that NREL researchers are working toward in their DFM development.

Besides reducing the ratio of hydrogen needed to produce methanol, another change that could make RCC more competitive with baseline low-carbon methanol production is simply time, as shown in Figure 4. Choosing the right location for initial pilot plants is also paramount, as shown in Figure 6. A location in the South Central US would seem to be ideal, with its high availability of both wind and solar resources, as well as nearby interests in developing renewable marine fuel for decarbonizing Gulf of Mexico shipping.

The ASPEN process model does not consider the effects of excess O_2_ and minor combustion products such as CO or NO in the flue gas. The ASPEN model merely recreates the measured CO_2_ adsorption capacity and methanol selectivity observed in the experiments. The model assumes that only CO_2_ can readily adsorb on the DFM catalyst and non-CO_2_ species will have no influence on CO_2_ adsorption. Additionally, the CO_2_ adsorption efficiency of this RCC process (in terms of % of the flue gas CO_2_ captured) has not been evaluated by experiments, necessitating a conservative estimate of 20% in the current TEA/LCA. Further development of the process model is needed to evaluate the true ceiling of RCC CO_2_ adsorption efficiency and maximize the amount of CO_2_ removed from the flue gas input stream. With input from our accompanying experimental effort, this development will also inform the CO_2_ adsorption capacity (in terms of mol CO_2_ absorbed per g catalyst) and methanol selectivity of the catalyst, with the goal to reduce the overall mass of the catalyst and size of the reactor. While reactor and catalyst costs and emissions were not found to be key components of the TEA/LCA, reducing the size and catalyst load of the RCC system and increasing its CO_2_ adsorption efficiency will increase the appeal of RCC as a retrofit option for existing CO_2_-producing plants and help stem the flow of CO_2_ emissions from these plants to the atmosphere.

The CZA catalyst load of the system will affect not just its cost, carbon emissions, and water consumption but also the consumption of three important metals: copper, zinc, and aluminum. In a 2023 Critical Materials Assessment by the U.S. Department of Energy (DOE),^35^ these three were among many materials studied for their importance to future supply chains. While zinc fell below DOE’s cutoff to be considered a “key material,” aluminum and copper were both assessed to be “near-critical” materials over the medium term (2025–2035), with copper having particularly high importance to energy and aluminum having a particularly high supply risk. Meanwhile, the size of the system will also affect the steel requirements for construction, steel which is currently mainly produced through carbon-intensive iron ore extraction and fossil fuel furnaces. Decarbonizing steel production is a key step in decarbonizing the entire industrial sector, and is another key DOE goal.^36^

Comparing the results from this work across renewable methanol TEA/LCA studies is difficult, since different sources of CO_2_ and H_2_ can yield wildly different results. A recent review of cradle-to-gate LCA studies of CO_2_-based methanol production, such as this one, found that CI results ranged from approximately −2 to 10 kg-CO_2_e/kg-MeOH^37^ (compared to the 0.55 kg-CO_2_e/kg-MeOH found herein for RCC methanol). This is why it was important to construct our “apples-to-apples” TEA/LCA model, which keeps the sources of CO_2_ and H_2_ consistent and changes only the methanol production process. There is similar variance in literature calculations of the cost of CO_2_-based methanol, and the International Renewable Energy Agency (IRENA) has published a useful compilation of these studies with estimated current costs and future costs.^9^ IRENA estimated costs of $0.82-$1.62 in 2015–2018 (compared to $0.99 for RCC), $0.41-$0.75 in 2030 (compared to $0.61 for RCC), and $0.25-$0.63 in 2050 (compared to $0.49 for RCC).

The unique feature of the model developed in this study is the ability to model an RCC methanol production process and guide further research in this area. We can see how RCC methanol production cost and emissions will vary with certain experimental parameters as well as technological developments, such as hydrogen electrolysis technology. Although the current experimental results do not show a price of RCC methanol that is below the baseline of CO_2_ hydrogenation methanol, we know the target H_2_:methanol ratio that must be achieved to accomplish this. Ongoing experiments at NREL with modified DFM catalysts are yielding promising results that will be combined with this TEA/LCA model (which in this paper was run using experimental results from an unmodified catalyst) in a forthcoming publication.

## Supporting Information Available

Description of the methods and sources used in the TEA and LCA; which was not included in the main article to meet length requirements (PDF)

## Abbreviations

AP: annual production
ATB: Annual Technology Baseline
BESS: battery energy storage system
CCS: carbon capture and storage
CCU: carbon capture and utilization
CCUS: carbon capture, utilization, and storage
CI: carbon intensity
CZA: Cu–Zn–Al
DFMs: dual function materials
FCR: fixed-charge rate
FOC: fixed operating cost
HOPP: Hybrids Optimization and Performance Platform
LC: levelized cost
LCA: life cycle assessment
LCOM: levelized cost of methanol
MeOH: methanol
NGCC: natural gas combined cycle
PEM: proton exchange membrane
PPA: power purchase agreement
RCC: reactive carbon capture
SAM: System Advisor Model
TCC: total capital cost
TEA: techno-economic analysis
TPSR: temperature and pressure swing reactor
VOC: variable operating cost
WC: water consumption
