# Supplementary material for: Temperature–Pressure Swing Process for Reactive Carbon Capture and Conversion to Methanol: Techno-Economic Analysis and Life Cycle Assessment
Source: Environ Sci Technol. 2024 Jul 24;58(31):13737–47. doi: 10.1021/acs.est.4c02589 (PMC11308513; doi:10.1021/acs.est.4c02589)
Supplement: Supplementary file 1 — es4c02589_si_001.pdf [file es4c02589_si_001.pdf]

Supporting Information

Paper Title: "A Temperature-Pressure-Swing Process for Reactive Carbon Capture and Conversion to Methanol – Techno-economic Analysis and Life Cycle Assessment"

Authors: Jonathan A. Martin[1]*, Eric C. D. Tan[1], Daniel A. Ruddy[1], Jennifer King[1], Anh T. To[1]

[1]National Renewable Energy Laboratory (NREL), 15013 Denver West Parkway, Golden, CO 80401, USA

*Corresponding author, email: jonathan.martin@nrel.gov

Number of Pages: 16

Figures:

Figure S-1. Correlation between MeOH reactor TOC and production capacity for baseline technology.

Figure S-2. Correlation between state-level Cambium electricity costs and AEO prices.

Figure S-3. Calculation of future wind electricity sales prices.

Figure S-4. Calculation of future solar electricity sales prices.

Figure S-5. Electricity purchases and sales to maintain high electrolyzer capacity

Figure S-6. Schematic breakdown of the baseline CO2 hydrogenation methanol production process.

Figure S-7. Schematic breakdown of the novel RCC (with recycle) methanol production process.

Tables:

Table S-1. Parameters for starting test cases.

Table S-2. Levelized costs evaluated in this study, and the various units used to report them

Table S-3. Conversion ratios

Table S-4. TEA sources and values used for Baseline CO2 Hydrogenation starting test case.

Table S-5. TEA sources and values used for RCC with recycle starting case.

Table S-6. LCA carbon intensity sources and values used for Baseline CO2 hydrogenation starting test case.

Table S-7. LCA water consumption sources and values used for Baseline CO2 hydrogenation starting test case.

Table S-8. LCA carbon intensity sources and values used for RCC with recycle starting test case.

Table S-9. LCA water consumption sources and values used for RCC with recycle starting test case.

Table S-10. Reactor performance parameter sweeps.

Table S-11. TEA and LCA results from the reactor performance sweeps.

SUPPORTING INFORMATION

All monetary values are in real terms, and have been converted from their source basis year (ranging between 2011 and 2020 in the various sources) to 2020 dollars based on the consumer price index (CPI).

Engineering constants used

Heating value of natural gas: 47.1 MJ/kg

Energy conversion: 1 MJ = 0.000948 MMBtu

Water volume conversion: 1 gal = 3.78 L, 1 L = 1 kg

(Used to convert Btu of NG to kg and gal of H_2O in source documents to kg, so all material flows are kg)

Starting Test Cases

All parameter sweeps in this study were based off of a set of starting parameters given below in Table S-1. For any parameter sweeps shown in the main paper, only one parameter is varied at a time from the set shown here.

Table S-1. Parameters for starting test cases. Some parameters will have different values for a non-recycle RCC plant than for a recycle RCC plant. The recycle case, which will produce more complete conversion of CO2 and H2 to methanol, is the basis for the modeled parameter sweeps.

Parameter	Unit	Baseline	NREL RCC (Non-recycle)	NREL RCC (Recycle)
Catalyst CO2 adsorption capacity	μmol/g-cat	N/A	66	66
Methanol productivity (single-pass)	μmol/g-cat	N/A	15.77	15.77
Methanol productivity (with recycle)	μmol/g-cat	N/A	N/A	68.13
Flue gas CO2 feed	tonnes/year	181,770	4,109,672	1,049,531
H2 consumed per unit methanol	g-H2/g-MeOH	0.195	1.002	0.327
Methanol product selectivity	%	N/A	23%	
Methanol production	t/y	115,104		
Methanol plant capacity factor	%	90% (7884 hr/y)		
Start-up year	year	2020		
Wind/solar/H2 hybrid site	Lat, Lon	32.34°, -98.27°		
NGCC plant location	Lat, Lon	32.34°, -97.73°		
	City, State	Granbury, TX		

Methods – TEA

The basis for evaluation in this study is real levelized cost (LC), i.e. the cost of *producing* energy or product, averaged over the lifetime of the plant, regardless of profit margins (hence not an actual *sales price* for the energy or product, which will be higher). Depending on the system component being evaluated, different forms of LC are evaluated. For electrical plants, such as the natural gas combined cycle (NGCC) plant, levelized cost of electricity (LCOE) is calculated in units of \$/MWh. However, the NGCC plant also generates CO_2 for the methanol processes, for which a levelized cost of CO_2 (LCOC) is calculated in units of \$/kg CO_2. Knowing the conversion rate of CO_2 to methanol in the methanol reactor, the LCOC can also be expressed in units of \$/kg MeOH. This allows the LCOC to be expressed

as a component of the overall cost of methanol, and compared with the cost of other components. Other levelized costs discussed include levelized cost of hydrogen (LCOH) and levelized cost of methanol (LCOM).

Table S-2 shows all the different forms of LC discussed in this Supporting Information, and the units used to report them.

Table S-2. Levelized costs evaluated in this study, and the various units used to report them

Plant component levelized costs	Product(s)	Cost	Primary Unit	Alternate Unit	Unit used to compare system-wide costs
NGCC without carbon capture	Electricity	LCOE	$/kWh_e$	$/MWh_e$	N/A
	Flue gas	LCOC	$/kg CO2		$/kg MeOH
NGCC **with** carbon capture	Electricity	LCOE	$/kWh_e$	$/MWh_e$	N/A
	CO2	LCOC	$/kg CO2		$/kg MeOH
Hybrid wind/solar	Electricity	LCOE	$/kWh_e$	$/MWh_e$	$/kg MeOH
PEM electrolyzer	H2	LCOH	$/kg H2	$/MMBTU	$/kg MeOH
All methanol plants	Methanol	LCOM	$/kg MeOH	$/MMBTU	$/kg MeOH

The individual component LCs can be converted into components of the overall levelized cost of methanol (LCOM), in units of $/kg MeOH, using the conversion ratios of each of the plants in these systems. The conversion ratios used in these plants, and sources for these values, are given below in Table S-3. The PEM H2 electrolyzer has a conversion ratio that shifts depending on the online year of the plant, modeling anticipated improvements in electrolyzer efficiency in the NREL H2A hydrogen production models [1].

Table S-3. Conversion ratios

Plant	Data Source	Conversion Ratios
NGCC plant without CO2 Capture	James et al. [2]	0.41637 kg NG = 1 kg CO2 + 2.9225 kWh$_e$
NGCC plant with CO2 Capture	James et al. [2]	0.46288 kg NG = 1 kg CO2 + 2.8860 kWh$_e$
PEM H2 Electrolyzer	James et al. [1]	54.66 kWh$_e$ + 14.32 kg H2O = 1 kg H2 (2020)<br>51.30 kWh$_e$ + 14.32 kg H2O = 1 kg H2 (2040)
Wind/solar hybrid + grid electricity	NREL HOPP Model [3]	X kWh$_e$ generated by hybrid + Y kWh$_e$ purchased from grid + Z* kWh$_e$ sold to grid + = 1 kWh$_e$ to electrolysis *SEE NOTE
CO2 Hydrogenation Reactor	Nyrari et al. [4]	1.423 kg CO2 + 0.195 kg H2 =<br>1 kg MeOH
NREL TPSR Reactors	Experiment [5] + ASPEN model (This Study)	9.118 kg CO2 + 0.327 kg H2 =<br>1 kg MeOH

*NOTE: X is calculated from hourly generation profiles from the wind/solar plant using NREL's HOPP software, then Y and Z are calculated on an hourly basis to meet an electrolyzer capacity factor of 97% (the capacity factor used in the H2A models).

The LCOE values calculated from the NGCC plants may seem irrelevant to LCOM, since their electricity product is outside the system boundaries in Figure 1 of the paper. However, the difference in LCOE between the plants with and without CO2 capture is needed to calculate LCOC for the baseline process. Since adding carbon capture to an existing NGCC plant will raise the LCOE, the difference in LCOE

between the plant with CC and the plant without CC will be the LCOC. This is the same process used to calculate the "breakeven CO2 sales price" in an NETL cost and performance baseline study [2]:

$$LCOC_{withCC} [\$/kg\ CO2] = (LCOE_{withCC} [\$/kWh] – LCOE_{withoutCC} [\$/kWh]) / CO2_{captured} [kg\ CO2/kWh] \quad (1)$$

The LCOC for the NGCC plant without carbon capture is zero since there are no modifications to the NGCC plant and the LCOE is not raised.

For all other system components, the LC of the product is calculated using a simple fixed-charge rate (FCR) method, based on the NREL System Advisor Model (SAM) [6]:

$$LC = (FCR * TCC + FOC) / AP + VOC \quad (2)$$

The other terms in equation (2) are:

- TCC: Total capital cost [$]
- FOC: Fixed operating cost [$/yr]
- AP: Annual production [MWh/yr, for energy OR mt/yr, for materials]
- VOC: Variable operating cost [$/MWh, for energy OR $/mt, for materials]

TCC, also referred to as total as-spent cost (TASC), is calculated from the total overnight cost (TOC), i.e. the total capital cost of building the component overnight, using the following equation:

$$TCC = TASC = TOC * TASC/TOC \quad (3)$$

The TOC values are enumerated from capital cost estimates of all the different parts of the system, and includes contractor services, contingencies, financing costs, etc. as outlined NETL's "Quality Guidelines for Energy System Studies (QGESS)" [7]. Since these capital costs are spread out over a capital expenditure period, a TASC/TOC multiplier must be applied to account for escalation and interest on debt. For this study, a constant 3-year capital expenditure period and 30 year recovery period were assumed, and thus a value of 1.093 for the TASC/TOC multiplier from the NETL QGESS was applied throughout the study. Likewise, a FCR of 7.07% was applied throughout the study, also taken from the NETL QGESS.

Combining equations (2) and (3) and entering the above constants produces a final equation for LC:

$$LC = (7.07\% * 1.093 * TOC + FOC) / AP + VOC \quad (4)$$

The sources and values for the four unknown terms in equations (4) are detailed in tables to follow. Most of these values can be directly taken from their sources or directly calculated from an equation in the source given in the tables to follow. However, three of these values could not be obtained from a single source alone, and were instead calculated from a combination of sources. Before giving the full tables detailing the sources of all the terms of equation (4), we will show how these three particular terms were calculated:

1. The TOC for the MeOH reactor component of the Baseline CO2 hydrogenation process
2. The positive VOC for electricity purchases from the grid to support H2 electrolysis
3. The negative VOC for electricity sales to the grid from excess wind/solar generation

The TOC for the baseline MeOH reactor was calculated by combining several literature TEA sources compiled by the International Renewable Energy Agency (IRENA) and the Methanol Institute in a 2021 report [8]. Cost estimates varied widely between studies but showed a trend of costs per unit of methanol produced going down with the size of the plant. For our baseline TEA, we made an exponential correlation of methanol production capacity to TOC per unit capacity using this data, seen in Figure S-1.

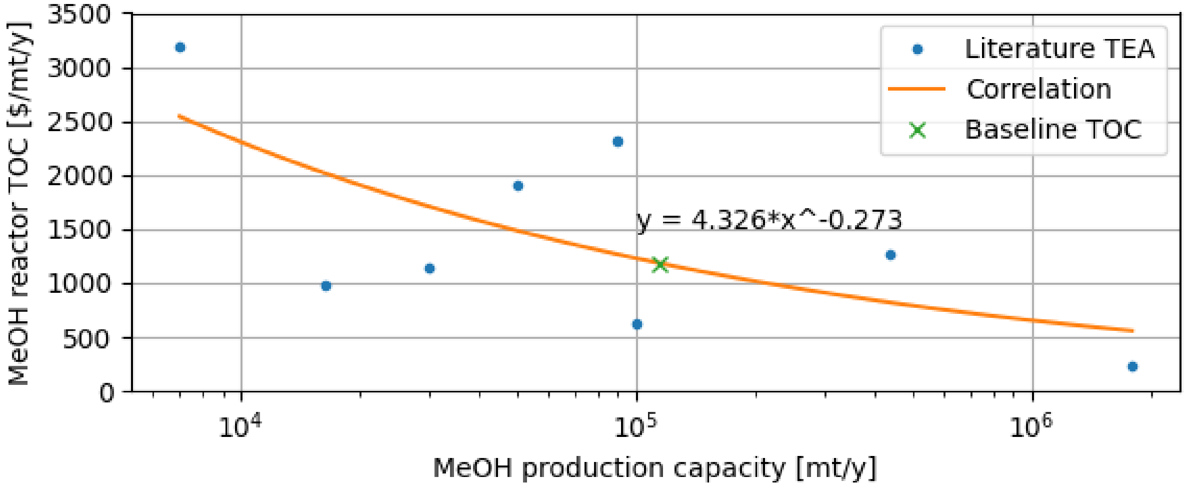

Figure S-1. Correlation between MeOH reactor TOC and production capacity for baseline technology.

To put a price on grid electricity purchases, state-level data from NREL's Cambium [9] grid modeling scenarios was used. Cambium combines several NREL tools to calculate the marginal increase in end use electricity costs caused by a local increase in demand. These costs are modeled out to 2050 using Cambium's "Mid-Case" scenario, which includes central estimates for inputs such as technology costs, fuel prices, and demand growth. However, the Cambium-calculated cost of electricity for did not match the cost of electricity as reported in the EIA's Annual Energy Outlook (AEO), as shown below in Figure S-2. Thus, the ratio between Cambium-calculated costs and AEO-measured prices for 2020 was calculated for each state, and used to calculate future electricity costs out to 2050. This is not a perfect estimate of future electricity costs, as local policies that cause differences between production costs and retail prices (such as overhead and administrative costs) may differ with time, but for the purposes of this paper we will assume that 2020 ratios between grid electricity price and production cost will remain in place. The expense of grid electricity compared to renewable wind and solar ensures that HOPP will design a plant that powers electrolysis primarily through wind and solar to minimize the LCOM.

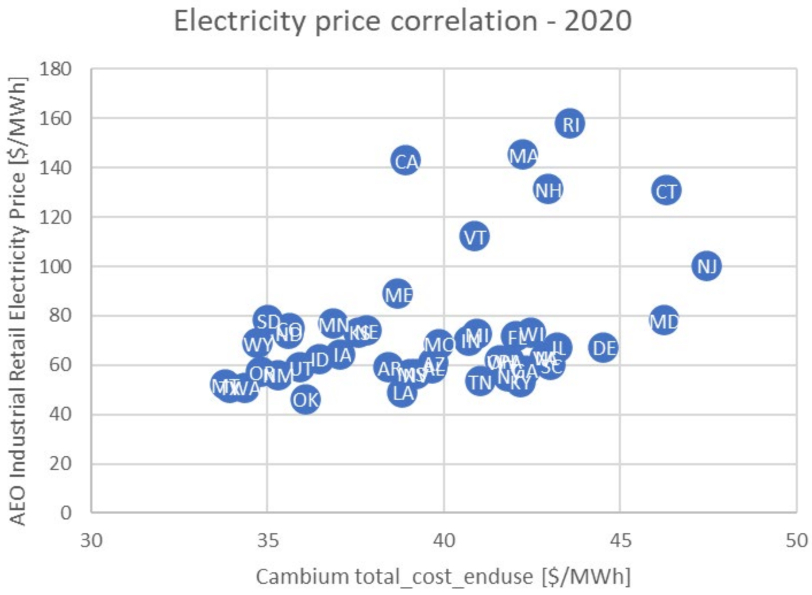

Figure S-2. Correlation between state-level Cambium electricity costs and AEO prices.

To put a price on grid electricity *sales* from excess renewable wind/solar production to the grid, a correlation was made between LCOE as calculated by the ATB and data on power purchase agreements (PPAs) collected by LBNL for wind [10] and solar [11] plants. These data show that PPA prices for renewable plants are typically slightly lower than the LCOE of generating the electricity. To project electricity sales prices, we assumed that the ratio between LCOE and PPA price will hold constant. First, a weighted average (based on plant MW capacity) of ppa prices between 2019 and 2021 was calculated, then divided by the LCOE as calculated by the ATB in 2020. This establishes a ratio of LCOE to electricity sales price that will be held constant across time and location, shown for wind in Figure S-3 and solar in Figure S-4.

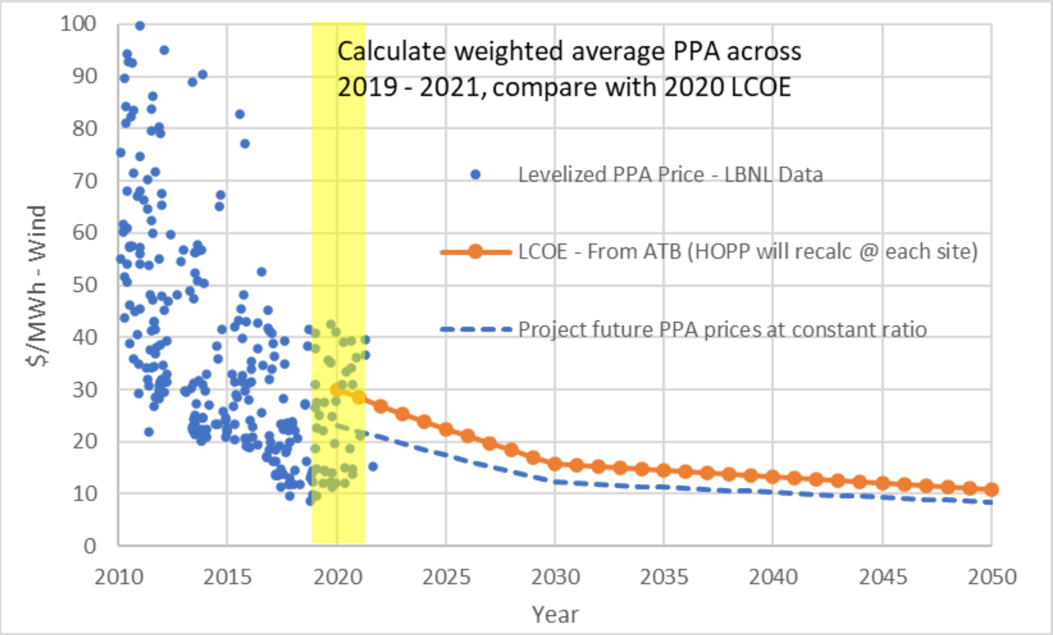

Figure S-3. Calculation of future wind electricity sales prices.

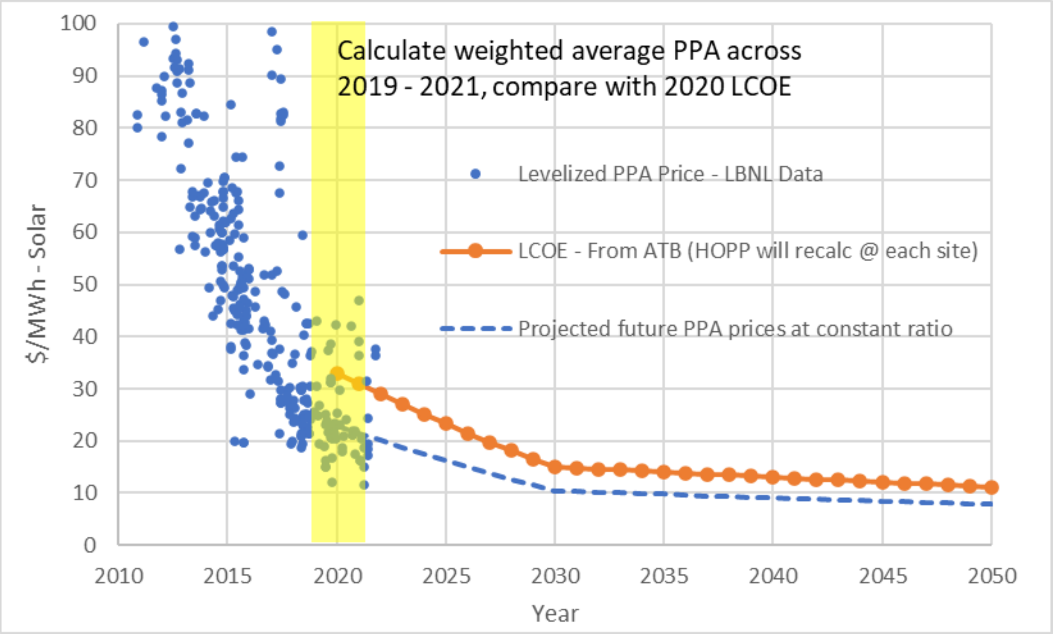

Figure S-4. Calculation of future solar electricity sales prices.

To determine how much grid electricity must be bought and excess generation sold at each location, HOPP is used to simulate plants' production over the course of a year using wind and solar resource data from the Wind Toolkit and National Solar Radiation Database. HOPP maintains electrolyzer output at 97% capacity to match the H2A scenarios and ensure minimal hydrogen production costs. Any generation over the electrolyzer capacity (144 MW in the baseline case) is sold, and if generation is under 94% capacity (136 MW in the baseline case), grid electricity is purchased to be able to maintain the electrolyzer at high capacity. This process is illustrated in Figure S-5.

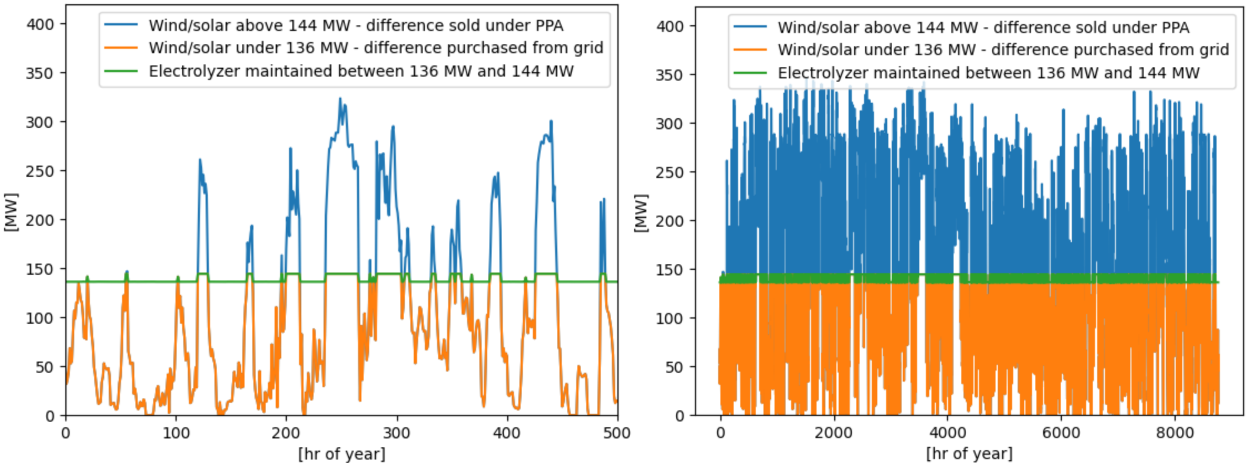

Figure S-5. Electricity purchases and sales to maintain high electrolyzer capacity

Once the total amount of each type of electricity (wind, solar, grid purchase, and grid sales) is tabulated, the final cost of electricity is calculated. By the same process, the final emission factors of the electricity used can be calculated, in which the NETL Grid Mix Explorer [12] assigns emissions factors to wind and solar generation and the NREL Cambium [9] model assigns end use marginal emissions factors to the grid electricity bought and sold, which vary from state to state depending on the electricity mix in each particular state. Electricity purchased counts for positive emissions, whereas electricity sold counts for negative emissions (the emissions of the grid electricity being *displaced* by the wind/solar electricity that is sold).

For all other values in the tables below, the costs could be modeled as constants or as much simpler lookup formulas from the sources listed. Some of these values are subject to change, based on the three parameter sweeps:

1. Reactor performance: Values that are depending on reactor performance are marked with a carrot^. These were based on experimental results [5], modeled at commercial scale in ASPEN.
2. Start-up year: Values that change depending on the year are marked with an asterisk*.
3. Location: Values that vary by geographic location are marked with a dagger†

For values that are subject to change, the values for the starting test cases are given in Table S-4 and Table S-5 below. Note that monetary values per unit mass are mostly given per metric ton (mt) instead of per kilogram to save on leading zeros. The highlighted values highlight anything that is a variable, i.e., anything that shifts away from these starting values throughout the course of the study due to parameter sweeps or HOPP optimization. Sources for all the values are noted in the tables.

Table S-4. TEA sources and values used for Baseline CO2 Hydrogenation starting test case.

**Baseline CO2 Hydrogenation (CO2)**	NGCC Plant w/o CO2 Capture	NGCC Plant with CO2 Capture	NG feed to both NGCC plants
TOC Source	NREL ATB [13]	NREL ATB [13]	N/A
TOC Value	$94.7 mil*	$213.2 mil*	N/A
FOC Source	NREL ATB [13]	NREL ATB [13]	N/A
FOC Value	$2,761k/yr	$5,853k/yr*	N/A
AP Source	100 MW_e Capacity, 85% C.F.	100 MW_e Capacity w/o CO2 capture	N/A
AP Value	744,600 MWh_e/yr	661,639 MWh_e/yr	N/A
VOC Source	NETL-PUB-22638 [2]	NETL-PUB-22638 [2]	NREL Cambium [9]
VOC Value	$1.83 /$MWh_e$	$5.94 /$MWh_e$	$119.74 /mt NG*†
Plant comp. of LCOE	$15.37 /$MWh_e$	$39.68 /$MWh_e$	
NG cost conversion ratio	0.41637 kg NG / 2.9225 kWh_e	0.46288 kg NG / 2.8860 kWh_e	
Fuel component of LCOE	$17.06 /$MWh_e$	$19.20 /$MWh_e$	
Total LCOE	$32.43 /$MWh_e$	$58.88 /$MWh_e$	
Conversion ratio from Table S-3	1 kg CO2 / 2.9225 kWh_e => $94.78 / mt CO2	1 kg CO2 / 2.8860 kWh_e => $169.93 / mt CO2	
**Total LCOC**	$169.93 - $94.78 = **$75.15 / mt CO2**		

**Baseline CO2 Hydrogenation (H2)**	PEM H2 electro-lyzer	Wind/ solar hybrid plant	Electricity purchase from grid	Electricity sales to grid
TOC Source	NREL H2A [1]	NREL ATB [13]	N/A	N/A
TOC Value	$86.2 mil*	$479.6 mil*	N/A	N/A
FOC Source	NREL H2A [1]	NREL ATB [13]	N/A	N/A
FOC Value	$4,885k /yr*	$13,692k /yr*	N/A	N/A
AP Source	Nyari et al. [4]	NREL HOPP	NREL HOPP	NREL HOPP
AP Value	22,445 mt/yr	1,281,485 MWh_e/yr†	351,987 MWh/yr†	406,613 MWh/yr†
VOC Source	NREL H2A	NREL ATB [13]	NREL Cambium [9] / EIA [14] / HOPP	LCOE + LBNL PPA data [11, 15]
VOC Value	$1.67 /mt H2	$0	$43.77 /$MWh_e$*†	-$30.35 /$MWh_e$*†
LC from Equation (4)	$516.09 /mt H2	$39.61 /$MWh_e$	$43.77 /$MWh_e$	-$30.35 /MWhe
Conversion ratio(s) from Table S-3	N/A	1.0445 kWh_e hybrid (=X) / 1 kWh_e to electrolysis†	0.28690 kWh_e from grid (=Y) / 1 kWh_e to electrolysis†	0.32898 kWh_e to grid (=Z) / 1 kWh_e to electrolysis†
Components of LCOE	N/A	$41.37 / MWh_e	$12.56 / MWh_e	-$9.98 / MWh_e
Total LCOE	N/A	$43.95 / MWh_e to electrolysis		
Conversion ratio from Table S-3	N/A	54.66 kWh_e / kg H2* => $2402.31 / mt H2		
**Total LCOH**	$516.09 + $2402.31 = **$2918.40 / mt H2**			

**Baseline CO2 Hydrogenation (MeOH)**	CO2 hydrogena-tion MeOH reactor	CO2 feed to MeOH reactor	H2 feed to MeOH reactor
TOC Source	IRENA report [8]	N/A	N/A
TOC Value	$136.0 mil	N/A	N/A
FOC Source	Nyari et al. [4]	N/A	N/A
FOC Value	$2,653k/yr	N/A	N/A
AP Source	Nyari et al. [4]	N/A	N/A
AP Value	115,104 mt/yr	N/A	N/A
VOC Source	Nyari et al. [4]	This table	This table
VOC Value	$0.79 /mt MeOH	$75.15 / mt CO2	$2918.40 /mt H2
LC from Equation (4)	$115.14 / mt MeOH	$75.15 / mt CO2	$2918.40 /mt H2
Conversion ratio from Table S-3	N/A	1.423 kg CO2 / 1 kg MeOH	0.195 kg H2/ 1 kg MeOH
Component of LCOM	$145.19 / mt MeOH	$106.94 / mt MeOH	$569.09 / mt MeOH
**Total LCOM**	$115.14 + $106.94 + $569.09 = **$791.17 / mt MeOH = $41.53 / MMBtu = $0.79 / kg MeOH**		

Table S-5. TEA sources and values used for RCC with recycle starting case.

**NREL RCC with Recycle (H2)**	PEM H2 electro-lyzer	Wind/ solar hybrid plant	Electricity purchase from grid	Electricity sales to grid	**NREL RCC with Recycle (MeOH)**	NREL pressure swing reactor	Flue gas feed to MeOH reactor (CO2)	H2 feed to MeOH reactor
TOC Source	NREL H2A [1]	NREL ATB [13]	N/A	N/A	TOC Source	NREL ASPEN Model	N/A	N/A
TOC Value	$117.4 mil*	$801.2 mil*	N/A	N/A	TOC Value	$33.3 mil^	N/A	N/A
FOC Source	NREL H2A [1]	NREL ATB [13]	N/A	N/A	FOC Source	NREL ASPEN Model	N/A	N/A
FOC Value	$6,167k /yr*	$22,870k /yr*	N/A	N/A	FOC Value	$1,529k/yr^	N/A	N/A
AP Source	Nyrai et al. [4]	NREL HOPP Model	NREL HOPP Model	NREL HOPP Model	AP Source	NREL ASPEN Model	N/A	N/A
AP Value	37,585 mt/yr	2,133,011† MWh_e/yr	591,304† MWh_e/yr	669,908† MWh_e/yr	AP Value	115,104 mt/yr	N/A	N/A
VOC Source	NREL H2A [1]	NREL ATB [13]	NREL Cambium [9] / EIA [14] / HOPP	LCOE + LBNL PPA data [11] [15]	VOC Source	NREL ASPEN Model	NGCC plant without CO2 capture	This table
VOC Value	$1.67 /mt H2	$0	$43.77*† /$MWh_e$	-$30.45*† /$MWh_e$	VOC Value	$35.81 /mt MeOH^	$0 / mt CO2	$2808.34 /mt H2
LC from Equation (4)	$407.13 /mt H2	$39.74 /$MWh_e$	$43.77 /$MWh_e$	-$30.45 /$MWh_e$	LC from Equation (4)	$71.45 / mt MeOH	$0 / mt CO2	$2808.34 /mt H2
Conversion ratio(s) from Table S-3	N/A	1.03826 kWh_e hybrid (=X) / 1 kWh_e to electrolysis	0.28782 kWh_e from grid (=Y) / 1 kWh_e to electrolysis	0.32608 kWh_e to grid (=Z) / 1 kWh_e to electrolysis	Conversion ratio from Table S-3	N/A	9.118 kg CO2 / 1 kg MeOH^	0.327 kg H2/ 1 kg MeOH^
Component of LCOE	N/A	$41.26 / MWh_e	$12.60 / MWh_e	-$9.93 / kWh_e	Component of LCOM	$71.45 / mt MeOH	$0 / mt MeOH	$918.33/ mt MeOH
Total LCOE	N/A	$43.93 / MWh_e to electrolysis			**Total LCOM**	$71.45 + $918.33 = **$989.78 / mt MeOH = $51.95 / MMBtu = $0.99 / kg MeOH**		
Conversion ratio from Table S-3	N/A	54.66 kWh_e / kg H2* => $2401.21 / mt H2						
**Total LCOH**	$407.13 + $2401.21 = **$2808.34 / mt H2**							

Methods – LCA

Similar to the tables for the TEA above, the tables below compile the sources and values used to calculate the carbon intensity (CI) and water consumption (WC) for the starting test cases of Baseline CO2 hydrogenation and NREL RCC with recycle, with highlights where values changed during parameter sweeps. These calculations were much more straightforward than the previous TEA calculations, except for hybrid wind/solar electricity emissions, which are explained above alongside the LCOE calculations.

Table S-6. LCA carbon intensity sources and values used for Baseline CO2 hydrogenation starting test case.

**Baseline CO2 Hydrogenation (CO2)**	NGCC Plant w/o CO2 Capture	NGCC Plant w/CO2 Capture	**Baseline CO2 Hydrogenation (H2)**	PEM H2 electrolyzer	Wind/ solar hybrid plant	Net electricity sales	**Baseline CO2 Hydrogenation (MeOH)**	CO2 hydrogenation MeOH reactor	CO2 feed	H2 feed
CI source	NETL LCA [10]	NETL LCA [10]	CI source	Zhao et al. [16]	NETL Grid Mix Explorer [12]/ HOPP	NREL Cam-bium [9]/ HOPP	CI source	Adnan and Kibria [17]	This table	This table
CI value	0.252 kg CO2e / kg CO2	0.392 kg CO2e / kg CO2	CI value	0.82 kg CO2e / MWh_e	28.78 kg CO2e / MWh_e*†	-3.71 kg CO2e / MWh_e*†	CI value, original units	0.020 kg CO2e / kg MeOH	0.140 kg CO2e / kg CO2	1.415 kg CO2e / kg H2
Total CI of CO2 capture	0.392-0.252 = **0.140 kg CO2e / kg CO2**		Total CI of H2 electro-lysis	0.82 + 28.78 – 3.71 = **25.89 kg CO2e / MWh_e** 54.66 kWh_e / kg H2 => **1.415 kg CO2e / kg H2**			Conver-sion ratio from Table S-3	N/A	1.423 kg CO2 / kg MeOH	0.195 kg H2/ kg MeOH
							CI value, kg CO2e/ kg MeOH	0.020	0.199	0.276
							**Grand total CI**	0.020 + 0.199 + 0.276 = **0.495 kg CO2e / kg MeOH**		

Table S-7. LCA water consumption sources and values used for Baseline CO2 hydrogenation starting test case.

**Baseline CO2 Hydrogenation (CO2)**	NGCC Plant w/o CO2 Capture	NGCC Plant w/CO2 Capture	**Baseline CO2 Hydrogenation (H2)**	PEM H2 electrolyzer	Wind/ solar hybrid plant	Net electricity sales	**Baseline CO2 Hydrogenation (MeOH)**	CO2 hydrogenation MeOH reactor	CO2 feed	H2 feed
WC source	NETL Grid Mix Explorer [12]/ NETL-PUB-22638 [2]		WC source	NREL H2A [1]	NETL Grid Mix Explorer [12]/ HOPP	NREL Cam-bium [9]/ HOPP	WC source	Nyrai et al. [4]	This table	This table
WC value	9.127 kg H2O / kg CO2	15.677 kg H2O / kg CO2	WC value	261.983 kg H2O / MWh_e	0.384 kg H2O / MWh_e*†	-20.407 kg H2O / MWh_e *†	WC value, original units	0.988 kg H2O / kg MeOH	6.550 kg H2O / kg CO2	13.226 kg H2O / kg H2
Total WC of CO2 capture	15.677-9.127 = **6.550 kg H2O / kg CO2**		Total WC of H2 electro-lysis	261.983 + 0.384 – 20.407 = **241.960 kg H2O / MWh_e** 54.66 kWh_e / kg H2 => **13.226 kg H2O / kg H2**			Conver-sion ratio from Table S-3	N/A	1.423 kg CO2 / kg MeOH	0.195 kg H2/ kg MeOH
							WC value, kg CO2e/ kg MeOH	0.988	9.321	2.579
							**Grand total WC**	0.988 + 9.321 + 2.579 = **12.888 kg H2O / kg MeOH**		

Table S-8. LCA carbon intensity sources and values used for RCC with recycle starting test case.

**RCC with recycle (H2)**	PEM H2 electrolyzer	Wind/ solar hybrid plant	Net electricity sales	**RCC with recycle (MeOH)**	RCC MeOH reactor	CO2 feed	H2 feed
CI source	Zhao et al. [16]	NETL Grid Mix Explorer [12]/ HOPP	NREL Cambium [9]/ HOPP	CI source	NREL ASPEN Model	N/A	This table
CI value	0.82 kg CO2e / MWh_e	28.78 kg CO2e / MWh_e*†	-3.42 kg CO2e / MWh_e*†	CI value, original units	0.078 kg CO2e / kg MeOH^	0.000 kg CO2e / kg CO2	1.431 kg CO2e / kg H2
Total CI of H2 electrolysis	0.82 + 28.78 – 3.42 =<br>**26.18 kg CO2e / MWh_e**<br>54.66 kWh_e / kg H2 =><br>**1.431 kg CO2e / kg H2**			Conversion ratio from Table S-3	N/A	9.118 kg CO2 / kg MeOH^	0.327 kg H2/ 1 kg MeOH^
				CI value, kg CO2e/ kg MeOH	0.078	0.000	0.468
				**Grand total CI**	0.078 + 0.000 + 0.468 =<br>**0.546 kg CO2e / kg MeOH**		

Table S-9. LCA water consumption sources and values used for RCC with recycle starting test case.

**RCC with recycle (H2)**	PEM H2 electrolyzer	Wind/ solar hybrid plant	Net electricity sales	**RCC with recycle (MeOH)**	RCC MeOH reactor	CO2 feed	H2 feed
WC source	Zhao et al. [16]	NETL Grid Mix Explorer [12]/ HOPP	NREL Cambium [9]/ HOPP	WC source	NREL ASPEN Model	N/A	This table
WC value	261.983 kg H2O / MWh_e	0.384 kg H2O / MWh_e*†	-18.734 kg H2O / MWh_e *†	WC value, original units	5.859 kg H2O / kg MeOH^	0.000 kg H2O / kg CO2	13.317 kg H2O / kg H2
Total WC of H2 electrolysis	261.983 + 0.384 – 18.734 =<br>**243.633 kg H2O / MWh_e**<br>54.66 kWh_e / kg H2 =><br>**13.317 kg H2O / kg H2**			Conversion ratio from Table S-3	N/A	9.118 kg CO2 / kg MeOH^	0.327 kg H2/ 1 kg MeOH^
				WC value, kg CO2e/ kg MeOH	5.859	0.000	4.355
				**Grand total WC**	5.859 + 0.000 + 4.355 =<br>**10.214 kg H2O / kg MeOH**		

To help visualize where exactly in the process these CO2 outputs and H2O inputs are coming from, schematic breakdowns of the baseline and RCC processes are given Figure S-6 and Figure S-7, respectively. Figure S-6 shows the entire baseline system, including the substantial water consumption needed for CO2 capture and purification to produce the pure CO2 supply for CO2 hydrogenation. This water consumption is an order of magnitude greater than the water consumption of the PEM electrolyzer, which was also supplied electricity by a wind/solar hybrid plant optimized by HOPP.

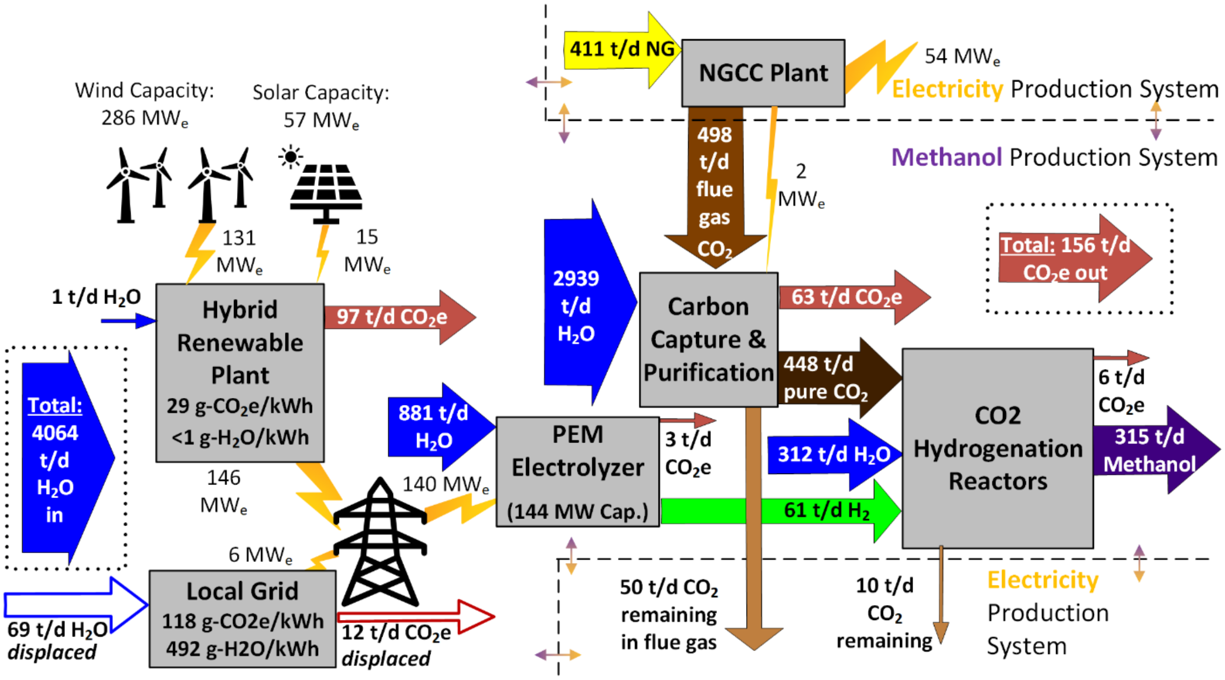

Figure S-6. Schematic breakdown of the baseline CO2 hydrogenation methanol production process.

It is important to note that the wind/solar hybrid's electrical output is not constant, but both the H2 electrolyzer and methanol plant must maintain a high capacity factor to be economical. This requires maintaining a grid connection so that grid electricity can be purchased when the wind solar/output is below the electrolyzer capacity, and excess wind/solar output can be sold back to the grid. HOPP makes sure the average output of the hybrid plant is equal to or greater than the input of the electrolyzer, so that the balance is a net export of clean electricity that displaces emissions from the local grid. This net export forms the negative "grid electricity displaced" bars seen on the bottoms of the stacked bar charts in Figures 2 and 3 in the main paper.

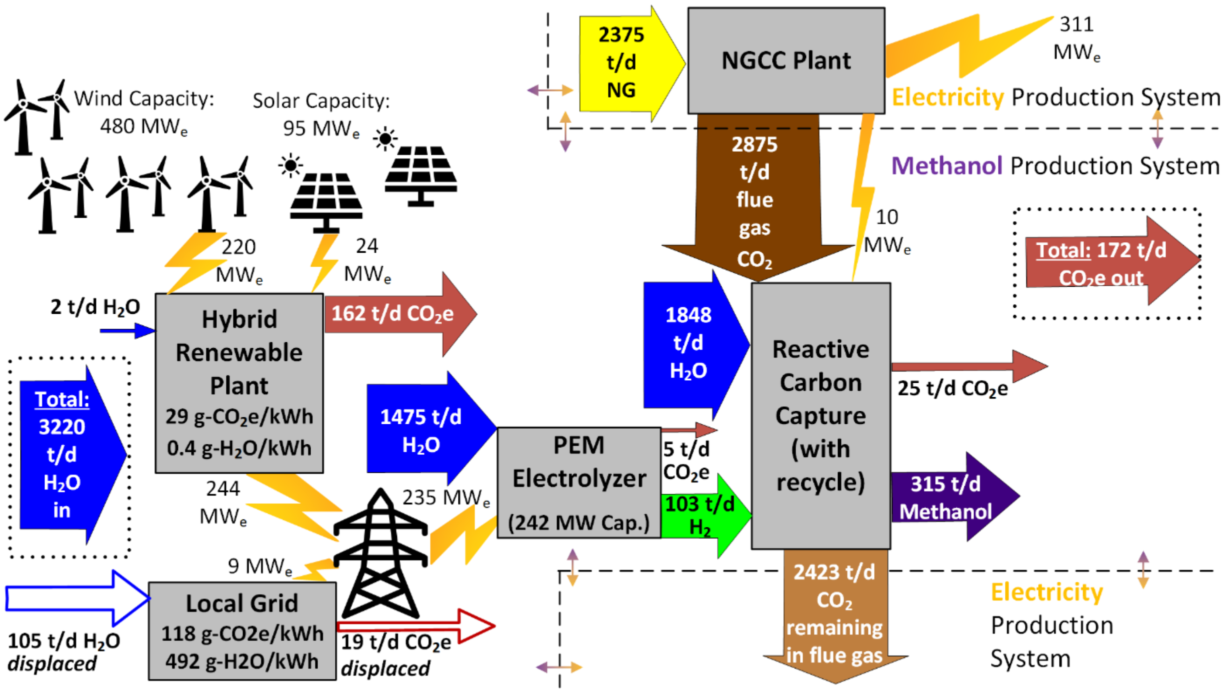

Figure S-7. Schematic breakdown of the novel RCC (with recycle) methanol production process.

The difference in the two processes is apparent by viewing Figure S-7 immediately after Figure S-6. The only constant between the two figures is the output of methanol, which is constant at 315 tonnes per day and matches the largest currently operating baseline methanol plant [8]. In Figure S-7, a larger hybrid wind/solar plant is needed to provide the greater hydrogen supply required by RCC, which generates greater indirect CO2 emissions than the hybrid plant in Figure S-6. A higher flowrate of flue gas CO2 is needed as well, which means that RCC cannot produce as much methanol from the same sized NGCC plant. On the other hand, the baseline process cannot capture and utilize as much carbon for the same cost. Additionally, in the current model, the RCC process off-gas containing H2, CO, and unrecovered methanol is sent to a boiler for steam generation; the co-product steam is sold to offset costs. For now, the next step being taken with the TEA/LCA modeling is to consider how to improve from the current results to make the RCC process favorable when compared to the baseline.

Results - Parameter Sweeps

The parameters for the full reactor performance sweeps are given below in Table S-10, with TEA and LCA results given below in Table S-11. Neither the CO2 adsorption capacity sweep nor the methanol selectivity sweep by themselves were able to produce LCOM or CI below the baseline. As shown in the paper, the target H2:methanol ratio of 0.26 is needed to achieve the LCOM and CI baseline.

Table S-10. Reactor performance parameter sweeps. The Starting Test Case is based on preliminary NREL experimental results

Sweep #	Parameter	Unit	Starting Test Case	Sweep Point # 1	Sweep Point # 2	Sweep Point # 3
1. CO2 adsorption capacity	**CO2 adsorption capacity**	**µmol/g-cat**	**66**	**94**	**122**	**150**
	Methanol selectivity	%	23%	23%	23%	23%
	H2 per unit methanol	g-H2/g-MeOH	0.33	0.33	0.33	0.33
2. Methanol selectivity	CO2 adsorption capacity	µmol/g-cat	66	66	66	66
	**Methanol selectivity**	**%**	**23%**	**32%**	**41%**	**50%**
	H2 per unit methanol	g-H2/g-MeOH	0.33	0.32	0.31	0.30
3. H2 per unit methanol	CO2 adsorption capacity	µmol/g-cat	66	66	66	66
	Methanol selectivity	%	23%	23%	23%	23%
	**H2 per unit methanol**	**g-H2/g-MeOH**	**0.33**	**0.28**	**0.24**	**0.2**

Table S-11. TEA and LCA results from the reactor performance sweeps.

	Baseline CO2 Hydrogenation	RCC starting test case	RCC Reactor Performance Sweep		
**CO_2 Adsorption Capacity, umol/g**	**-**	**66**	**94**	**122**	**150**
LCOM, \$/kg-MeOH	\$0.79	\$0.99	\$0.96	\$0.94	\$0.93
CI, kg-CO_2/kg-MeOH	0.495	0.546	0.523	0.510	0.502
WC, kg-H_2O/kg-MeOH	10.833	6.767	6.722	6.698	6.683
**Methanol Selectivity, mass % of single-pass**	**-**	**23**	**32**	**41**	**50**
LCOM, \$/kg-MeOH	\$0.79	\$0.99	\$0.97	\$0.94	\$0.92
CI, kg-CO_2/kg-MeOH	0.495	0.546	0.530	0.510	0.499
WC, kg-H_2O/kg-MeOH	10.833	6.767	6.273	5.778	5.289
**H_2:Methanol ratio, kg-H_2/kg-MeOH**	**0.20**	**0.33**	**0.28**	**0.24**	**0.20**
LCOM, \$/kg-MeOH	\$0.79	\$0.99	\$0.87	\$0.76	\$0.65
CI, kg-CO_2/kg-MeOH	0.495	0.546	0.480	0.422	0.365
WC, kg-H_2O/kg-MeOH	12.889	10.214	9.588	9.055	8.523

References

(1) James, B.; Colella, W.; Moton, J.; Saur, G. a. R. T. *PEM Electrolysis H2A Production Case Study Documentation*; NREL/TP-5400-61387; National Renewable Energy Laboratory: Golden, CO, 2013; DOI 10.2172/1214980.

(2) James, I. R. E.; Keairns, D.; Turner, M.; Woods, M.; Kuehn, N.; Zoelle, A. *Cost and Performance Baseline for Fossil Energy Plants Volume 1: Bituminous Coal and Natural Gas to Electricity*; NETL-PUB-22638; National Energy Technology Laboratory: Pittsburgh, PA, 2019; DOI 10.2172/1569246.

(3) Clark, C. E.; Barker, A.; King, J.; Reilly, J. *Wind and Solar Hybrid Power Plants for Energy Resilience*; NREL/TP-5R00-80415; National Renwable Energy Laboratory: Golden, CO, 2022; DOI 10.2172/1842446.

(4) Nyári, J.; Magdeldin, M.; Larmi, M.; Järvinen, M.; Santasalo-Aarnio, A. Techno-economic barriers of an industrial-scale methanol CCU-plant. *Journal of CO2 utilization* **2020**, *39*, 101166, DOI 10.1016/j.jcou.2020.101166.

(5) Jeong-Potter, C.; Arellano-Treviño, M. A.; McNeary, W. W.; Hill, A. J.; Ruddy, D. A.; To, A. T. Modified Cu–Zn–Al mixed oxide dual function materials enable reactive carbon capture to methanol. *EES Catalysis* **2024**, DOI 10.1039/D3EY00254C.

(6) Freeman, J. M.; DiOrio, N. A.; Blair, N. J.; Neises, T. W.; Wagner, M. J.; Gilman, P.; Janzou, S. *System advisor model (SAM) general description (version 2017.9. 5)*; NREL/TP-6A20-70414; National Renewable Energy Laboratory: Golden, CO, 2018, DOI 10.2172/1440404.

(7) Theis, J. *Quality Guidelines for Energy Systems Studies: Cost Estimation Methodology for NETL Assessments of Power Plant Performance*; NETL-PUB-22580; National Energy Technology Laboratory: Pittsburgh, PA, 2021, DOI 10.2172/1567736.

(8) IRENA and Methanol Institute, *Innovation Outlook: Renewable Methanol*; International Renewable Energy Agency: Abu Dhabi, 2021, www.irena.org/-/media/Files/IRENA/Agency/Publication/2021/Jan/IRENA_Innovation_Renewable_Methanol_2021.pdf

(9) Gagnon, P.; Cowiestoll, B.; Schwarz, M. *Cambium 2022 Scenario Descriptions and Documentation*; NREL/TP-6A40-84916; National Renewable Energy Laboratory: Golden, CO, 2022, DOI 10.2172/1915250.

(10) Skone, T. J.; Schivley, G.; Jamieson, M.; Marriott, J.; Cooney, G.; Littlefield, J.; Mutchek, M.; Krynock, M.; Shih, C. Y. *Life Cycle Analysis: Natural Gas Combined Cycle (NGCC) Power Plants*; DOE/NETL-2018/1890; National Energy Technology Laboratory: Pittsburgh, PA, 2018; DOI 10.2172/1562914.

(11) Bolinger, M.; Seel, J.; Warner, C.; Robson, D. *Utility-Scale Solar, 2022 Edition: Empirical Trends in Deployment, Technology, Cost, Performance, PPA Pricing, and Value in the United States*; Lawrence Berkeley National Lab: Berkeley, CA, 2022; DOI 10.2172/1888246.

(12) Skone, T. J. *Grid Mix Explorer Version 4*; National Energy Technology Laboratory: Pittsburgh, PA, 2019, www.osti.gov/biblio/1580052.

(13) Vimmerstedt, L.; Stehly, T.; Akar, S.; Sekar, A.; Mirletz, B.; Stright, D.; Augustine, C.; Beiter, P.; Bhaskar, P.; Blair, N.; Cohen, S.; Cole, W.; Duffy, P.; Feldman, D.; Gagnon, P.; Kurup, P. *2022 Annual Technology Baseline (ATB) Cost and Performance Data for Electricity Generation Technologies*; National Renewable Energy Laboratory: Golden, CO, 2022; DOI 10.25984/1871952.

(14) US Energy Information Administration. Average retail price of electricity, annual. www.eia.gov/electricity/data.php (accessed March 22, 2023).

(15) Wiser, R.; Bolinger, M.; Hoen, B.; Millstein, D.; Rand, J.; Barbose, G.; Darghouth, N.; Gorman, W.; Jeong, S.; Paulos, B. *Land-Based Wind Market Report: 2022 Edition*; Lawrence Berkeley National Lab: Berkeley, CA, 2022; DOI 10.2172/1882594.

(16) Zhao, G.; Kraglund, M. R.; Frandsen, H. L.; Wulff, A. C.; Jensen, S. H.; Chen, M.; Graves, C. R. Life cycle assessment of H2O electrolysis technologies. *International Journal of Hydrogen Energy* **2020**, *45* (43), 23765-23781, DOI 10.1016/j.ijhydene.2020.05.282.

(17) Adnan, M. A.; Kibria, M. G. Comparative techno-economic and life-cycle assessment of power-to-methanol synthesis pathways. *Applied Energy* **2020**, *278*, 115614, DOI 10.1016/j.apenergy.2020.115614.
